# Molecular Analysis of an Outbreak of Lethal Postpartum Sepsis Caused by Streptococcus pyogenes

**DOI:** 10.1128/JCM.00679-13

**Published:** 2013-07

**Authors:** Claire E. Turner, Matthew Dryden, Matthew T. G. Holden, Frances J. Davies, Richard A. Lawrenson, Leili Farzaneh, Stephen D. Bentley, Androulla Efstratiou, Shiranee Sriskandan

**Affiliations:** Infectious Diseases & Immunity, Imperial College London, Hammersmith Hospital, London, United Kingdoma; Royal Hampshire County Hospital, Winchester, Hampshire, United Kingdomb; Pathogen Genomics, The Wellcome Trust Sanger Institute, Hinxton, Cambridge, United Kingdomc; Microbiology Reference Services Division, Public Health England, Colindale, London, United Kingdomd

## Abstract

Sepsis is now the leading direct cause of maternal death in the United Kingdom, and Streptococcus pyogenes is the leading pathogen. We combined conventional and genomic analyses to define the duration and scale of a lethal outbreak. Two postpartum deaths caused by S. pyogenes occurred within 24 h; one was characterized by bacteremia and shock and the other by hemorrhagic pneumonia. The women gave birth within minutes of each other in the same maternity unit 2 days earlier. Seven additional infections in health care and household contacts were subsequently detected and treated. All cluster-associated S. pyogenes isolates were genotype *emm*1 and were initially indistinguishable from other United Kingdom *emm*1 isolates. Sequencing of the virulence gene *sic* revealed that all outbreak isolates had the same unique *sic* type. Genome sequencing confirmed that the cluster was caused by a unique S. pyogenes clone. Transmission between patients occurred on a single day and was associated with casual contact only. A single isolate from one patient demonstrated a sequence change in *sic* consistent with longer infection duration. Transmission to health care workers was traced to single clinical contacts with index cases. The last case was detected 18 days after the first case. Following enhanced surveillance, the outbreak isolate was not detected again. Mutations in bacterial regulatory genes played no detectable role in this outbreak, illustrating the intrinsic ability of *emm*1 S. pyogenes to spread while retaining virulence. This fast-moving outbreak highlights the potential of S. pyogenes to cause a range of diseases in the puerperium with rapid transmission, underlining the importance of immediate recognition and response by clinical infection and occupational health teams.

## INTRODUCTION

Cases of postpartum maternal Streptococcus pyogenes sepsis occur sporadically; paired cases and deaths are rare in the developed world (1, 2). We describe two postpartum deaths due to S. pyogenes (group A Streptococcus [GAS]) that occurred within 24 h in the same maternity unit; one case was characterized by septic shock and the other by hemorrhagic pneumonia. Both cases were associated with seven additional infections detected in health care and family contacts. We combined genotypic and phenotypic analyses with whole-genome sequencing to confirm that the isolates were unique and distinct from other *emm*1 GAS isolates circulating in the United Kingdom, enabling us to determine the time limits and scale of this outbreak.

## CASE REPORTS

### Case 1.

A 39-year-old teacher (gravida 3, para 2) presented at term with irregular contractions and was monitored for 5 h in the maternity unit prior to discharge. She was readmitted 5 h later and delivered a live female infant. Other than a small second-degree tear, there were no complications and the mother was discharged 13 h after delivery. Approximately 30 h following delivery she awoke with lower abdominal pain necessitating hospital readmission, inhaled nitrous oxide, and opiates, although physiological measurements were initially normal. She collapsed, 6 h later, with hypotension and was transferred to the intensive care unit. She rapidly developed multiorgan failure and responded poorly to inotropic and ventilatory support in addition to broad-spectrum antibiotics. The following day, blood cultures indicated a streptococcal infection, and high-dose intravenous pooled human immunoglobulin G (IVIG) was administered. The patient deteriorated and died 2.5 days after delivery of her third child. GAS was isolated from blood cultures and genital tract swabs obtained antemortem and also from uterine cervix tissue obtained postmortem.

### Case 2.

A 29-year-old primigravid teacher was admitted while in labor to the same unit on the same day as case 1. She had experienced spontaneous rupture of membranes 48 h earlier and had commenced prophylactic amoxicillin. During the day she had had a transient temperature of 37.7°C which settled, though other physiological measurements were normal. An oxytocin infusion was commenced; 12 h later, a live male infant was delivered by emergency cesarean section, and a final antibiotic dose was administered intraoperatively. The time of cesarean delivery was 2 min before the delivery of the baby of case 1, although the delivery occurred in a separate section of the maternity unit and was undertaken by different staff. She was discharged approximately 66 h after cesarean section. Approximately 76 h after delivery, she developed a cough and chest pain associated with blood-stained sputum. A community midwife arranged hospital readmission. While packing, the patient collapsed in respiratory arrest associated with hemoptysis. Resuscitation was attempted by a paramedical team on site and in the hospital emergency unit but the patient died on 24 December, 3.5 days after cesarean section. GAS was isolated from throat, hemorrhagic lung, and uterine cervix samples postmortem but not from the cesarean wound or lower genital tract. GAS isolates from both cases were subsequently identified as *emm* type 1.

### Response of infection team and intensive surveillance.

**Fig 1:**
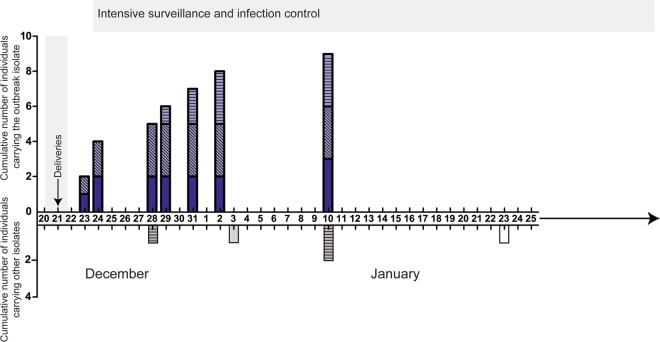
Identification of group A streptococcus (GAS) cases. Cumulative number of individuals carrying or infected with the outbreak strain by date (upper axis, blue-shaded bars). Individuals with the outbreak strain included maternity patients (solid shading), household contacts (diagonal crosshatching), and health care workers (HCWs) (horizontal crosshatching). Individuals found to be carrying or infected with other GAS isolates during the period of surveillance are shown on the lower axis (gray-shaded bars). Period of overlapping hospital admissions for three maternity patients denoted by gray shaded area. During the period of intense surveillance, which started on December 24 and lasted for 2 months, two HCWs were identified to be carrying *emm*12 GAS (gray-shaded horizontally hatched bars) and two nonmaternity patients had community-acquired invasive infections caused by GAS strains *emm*89 (gray-shaded bar, January 3) and *emm*75 (white bar, January 23). Enhanced surveillance continued for a further 4 months, during which time no further GAS was isolated from any maternity patient.

A hospital outbreak control team managed the incident, surveillance, and epidemiology. Household and health care worker (HCW) contacts of each patient were screened, including antenatal, during-labor, and postnatal contacts (Fig. 1). Environmental screening was conducted once in the immediate aftermath of the outbreak by swabbing of baths, showers, basins, and bed space surfaces, and the samples obtained by swabbing were cultured on blood agar.

As part of a 2-month period of intensive surveillance, all mothers who had been on the unit concurrently with the two cases were contacted and advised to seek medical attention and screening if symptomatic. All women in labor were screened during this period within a day of admission. Throat and skin swabs (if skin lesions were reported) were used for screening for GAS, plus lower genital tract swabs for all women in labor. During the intensive surveillance period, enhanced twice daily chlorine-based cleaning in the maternity unit was undertaken (previously once daily detergent-based cleaning).

### Screening results. (i) Household contacts.

Four household contacts of case 1 were screened, of whom 2 were positive for GAS. Two household contacts of case 2 were screened, of whom 1 was positive for GAS.

The baby of case 1 was admitted to the neonatal unit with a raised level of C-reactive protein (CRP) and received ceftriaxone for suspected sepsis; nose and ear swabs obtained 2 days after delivery yielded GAS *emm*1, although blood and cerebrospinal fluid (CSF) cultures were negative. The baby of case 2 was not colonized or infected with GAS.

Throat swabs from the partners of case 1 and case 2 yielded GAS *emm*1, and both of these individuals received antibiotics. One partner reported pharyngitis at the time of screening (i.e., after the death of case 1), while the other developed a lower respiratory tract infection necessitating hospital admission 4 days after the death of case 2.

### (ii) Health care contacts.

Of 69 HCW contacts screened, 3/69 (4.3%) had throat swabs that yielded GAS *emm*1, and each received antibiotic treatment. The three HCWs reported single episodes of postnatal clinical contact with the patients and included an obstetrician who examined case 1, an intensive care unit (ICU) nurse present at the intubation of case 1, and a community midwife attending case 2 at home. Two of the HCWs developed significant symptoms 7 to 10 days after the deaths; one had tonsillitis and cervical lymphadenopathy, while the other had pharyngitis. HCWs who tested positive for GAS were offered 10 days of treatment with oral amoxicillin, removed from work for this period, and screened again at the end of treatment. The partner of a symptomatic HCW was treated for GAS pharyngitis, though the isolate was not available for study.

One hundred ninety-three HCWs from the same institution, who were not contacts of the two cases, were also screened. Two of the 193 (1%) had throat swabs that yielded GAS and received antibiotics; however, these isolates were *emm*12.

### (iii) Other patients.

In the immediate aftermath of the outbreak, 42 maternity patients were screened, including women who reported symptoms compatible with GAS and had been on the unit at the same time as the two fatal cases, along with those admitted up to 2 weeks later. One of the 42 had a throat swab that yielded GAS, typed as *emm*1. This patient reported pharyngitis and sinusitis that developed 1 week postpartum and also recalled meeting and talking with case 1 on the day of her original admission (Fig. 1). This patient was treated with antibiotics in the community.

Two further cases of invasive GAS infection (necrotizing fasciitis and pneumonia) were admitted from the community during the month following the maternal fatalities; however, the GAS isolates in these cases were *emm*75 and *emm*89 (Fig. 1).

### (iv) Environment.

GAS was not isolated from any environmental samples.

### (v) Enhanced surveillance.

A further 4-month period of enhanced clinical surveillance was instituted within the obstetric and neonatal units, in which HCW were particularly alert for all GAS infections and swabbed throats and skin of patients or HCW if there was any history or indication of inflammation. No further GAS carriage or infection was detected.

## MATERIALS AND METHODS

### Phenotyping and genotyping of GAS strains.

All 15 *emm*1 GAS isolates from the cluster were compared with 14 other *emm*1 GAS isolates submitted to the national reference laboratory from elsewhere in England in the 3 months preceding and 1 month following the outbreak, including, where available, isolates from the same region (see Table S1 in the supplemental material). GAS phenotypic comparison, *emm* typing, superantigen genotyping, and *covR/S* and *sic* loci sequencing are described in Supplementary Methods in the supplemental material.

### GAS whole-genome sequencing.

Illumina sequencing was performed on 24 strains, including all 15 strains from the outbreak, and mapped against the complete genome sequence of the U.S. *emm*1 strain MGAS5005 (3). In excess of 50-fold sequence coverage was achieved for all isolates (see Supplementary Methods in the supplemental material).

### Measurement of anti-*emm*1 GAS immunity in pregnant women.

As a surrogate for immunity among women of child-bearing age in the United Kingdom, anonymized serum samples from antenatal screening were obtained from the Imperial College Healthcare Trust and used in accordance with approval from the West London Research Ethics Committee.

### Enzyme-linked immunosorbent assay-based assays of immunity.

Reactivity of antenatal sera to recombinant SIC proteins was tested using an enzyme-linked immunosorbent assay (ELISA)-based method. Diluted antenatal sera were applied to SIC protein-coated wells, and bound IgG was detected with goat anti-human IgG-horseradish peroxidase (HRP). In order to quantify SIC-reactive IgG in each serum sample relative to the human blood product intravenous immunoglobulin (IVIG) (Zenalb 4.5), which has high antistreptococcal activity, the activity of IVIG against SIC protein was also measured using a series of fixed concentrations of IVIG to generate a standard curve of IVIG-equivalent anti-SIC IgG on each plate. A similar protocol was used to detect IgG reactivity of antenatal sera to whole *emm*1 S. pyogenes cells, but the wells were coated with heat-killed *emm*1 S. pyogenes. Details are provided in the supplemental methods in the supplemental material.

### Opsonophagocytosis assays of immunity.

Assays were performed using 80 (of 199) representative heat-inactivated antenatal patient sera. *emm*1 GAS (strain H584) isolates were stained with fluorescein isothiocyanate (FITC) (Invitrogen) and opsonized with test serum before being added to 2 × 10^6^ fresh human neutrophils and 10% complement (rabbit serum; Merck Chemicals, United Kingdom). Neutrophils with internalized FITC-labeled bacteria were measured by flow cytometry. To exclude the external adherent, FITC-labeled S. pyogenes, samples were quenched with trypan blue. Antenatal serum-opsonized GAS isolates were compared with IVIG (2.5 mg/ml)-opsonized GAS isolates, as this has been shown to provide optimum opsonophagocytosis. Nonopsonized GAS isolates were used as negative controls.

## RESULTS

### Phenotypic analysis of GAS isolates.

To determine whether the 15 *emm*1 GAS isolates from the maternity unit exhibited excessive virulence, they were compared phenotypically with 14 other *emm*1 strains from the United Kingdom using a wide panel of *in vitro* analyses that included investigations of growth in whole blood, superantigenicity, and the expression of capsule, Streptococcus pyogenes cell envelope protease (SpyCEP), and cysteine protease SPEB (see Fig. S1 in the supplemental material). There were no phenotypic differences detected between the outbreak cluster and other *emm*1 strains circulating in the United Kingdom. Isolates were penicillin sensitive, and the MIC did not differ from other *emm*1 isolates (0.03 mg/liter).

### Molecular analysis of GAS isolates.

Although 5 to 10 different superantigen genotypes have been described in European *emm*1 strains (4), all 29 outbreak and nonoutbreak strains had the same superantigen genotype (*speA*, *speG*, *speJ*, and *smeZ*).

Sequencing of the streptococcal inhibitor of complement (SIC), encoded by *sic*, identified 13 different *sic* alleles among all 29 *emm*1 isolates studied, the most common allele identified being *sic1.02* (see Table S1 in the supplemental material). Of the 15 *emm*1 isolates from the maternity unit, 14 had the same *sic* gene, which had a unique gene sequence and was designated *sic1.300*. One genital tract isolate from case 1 demonstrated another unique *sic* allele, designated *sic1.301*. As *sic1.301* differed from *sic1.300* by an 87-bp deletion (see Fig. S2 in the supplemental material), we surmised that *sic1.301* had arisen from *sic1.300* during mucosal carriage indicating possible longer duration of infection (5, 6).

Despite distinctive sequences, there were no differences in the abilities of the recombinant variant SIC proteins to inhibit complement-mediated erythrocyte lysis (SIC1.300, SIC1.301, and the common United Kingdom type SIC1.02) (see Fig. S2 in the supplemental material), a finding that is consistent with those reported in published structure-function data (5, 7).

### Whole-genome sequencing.

To determine if any features in addition to those revealed by the *sic* genotyping distinguished the isolates, we performed whole-genome sequencing of the 15 *emm*1 isolates from the maternity unit and 9 of the 14 unrelated *emm*1 isolates from elsewhere in England.

**Fig 2:**
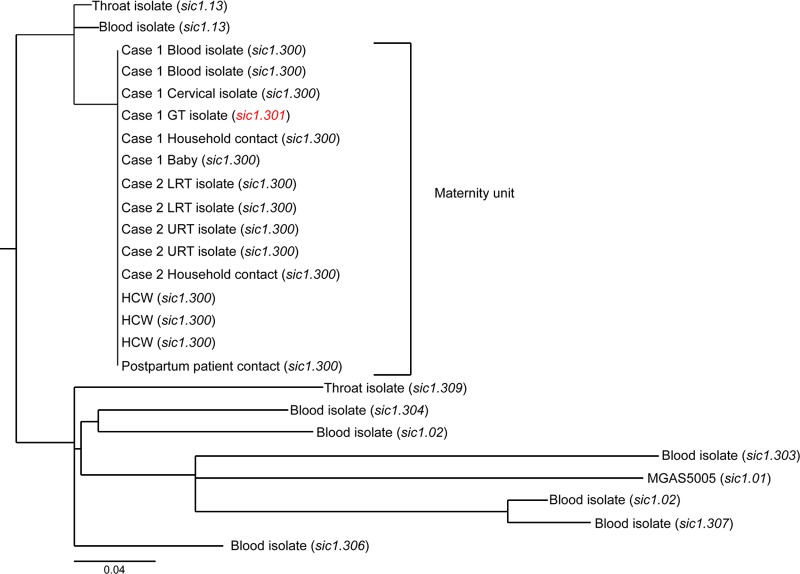
Phylogenetic analysis results demonstrating that the core genomes of the maternity unit isolates were identical to each other but different from contemporaneous *emm*1 isolates submitted to the reference laboratory. The genomes of all 15 isolates from the maternity unit and 9 *emm*1 isolates from around England were sequenced and mapped to the complete genome sequence of MGAS5005, a contemporary U.S. strain (3). A maximum-likelihood phylogenetic tree was generated from core genome single nucleotide polymorphisms (SNPs) of 24 *emm*1 isolates. The maternity unit isolates clustered in a single clade that differed from the nearest relative by eight SNPs. The *sic* allele for each isolate is indicated. The unique *sic* allele (*sic1.301*) that arose within the outbreak cluster is highlighted in red. The scale bar represents substitutions per SNP site. Details of all SNPs are provided in Table S2 in the supplemental material. HCW, health care worker; URT, upper respiratory tract; LRT, lower respiratory tract; GT, genital tract.

Genome sequencing confirmed that the 15 strains from the maternity unit were unique and differed from the nearest GAS relative by eight core genome single nucleotide polymorphisms (SNPs); six of these SNPs were unique to the outbreak cluster (Fig. 2). Of the six unique SNPs, one was nonsynonymous and occurred in the *sic* gene (consistent with the previously noted unique *sic* gene sequence). Additionally, outside the core genome, the outbreak cluster had a unique nonsynonymous SNP that mapped to a gene of unknown function in phage 5005.2.

Comparing all 24 sequenced *emm*1 strains with the genome of MGAS5005, the most recently sequenced U.S. *emm*1 strain (3), we identified a total of 250 SNP loci within the core genome (excluding phage-related sequences), of which 138 (∼55%) were nonsynonymous substitutions, 80 (∼32%) were synonymous substitutions, and 32 (∼13%) were within intergenic regions (see Tables S2, S3, and S4 in the supplemental material).

Mutations in GAS regulatory genes, reported to be associated with strain hyperinvasiveness, were not seen in any of the 15 isolates from the maternity unit. Unique mutations in *covR/S* and *rgg* were seen in only 2/7 and 1/7 invasive *emm*1 strains from elsewhere in England, respectively, and these were associated with predicted phenotypic changes that included increased SpyCEP and capsule expression but reduced SPEB expression (see Fig. S1 in the supplemental material).

During the short outbreak course, no new SNPs arose in the 15 isolates from the maternity unit. However, in addition to the *sic* deletion mutation already detected in a genital tract isolate from case 1, a truncation deletion mutation in the capsule biosynthesis gene, *hasB*, was identified in a pharyngeal isolate cultured from the maternity patient contact with an 11-day history of pharyngitis. This mutation had no measurable impact on capsule production *in vitro* (see Fig. S1 in the supplemental material).

### Immunity to *emm*1 GAS in antenatal population.

To investigate antenatal population immunity to *emm*1 GAS, 199 sera were tested for reactivity to each SIC allele (*sic* being specific to *emm*1) and to *emm*1 GAS surface proteins. Of the 199 sera, 7% showed no recognition of SIC, while a majority showed low reactivity and a small proportion (8%) showed strong reactivity similar to IVIG. SIC allele-specific responses were seen only in a minority (see Fig. S3 in the supplemental material). Only 16% of the 199 antenatal sera had any detectable antibody to GAS surface proteins (see Fig. S4).

**Fig 3:**
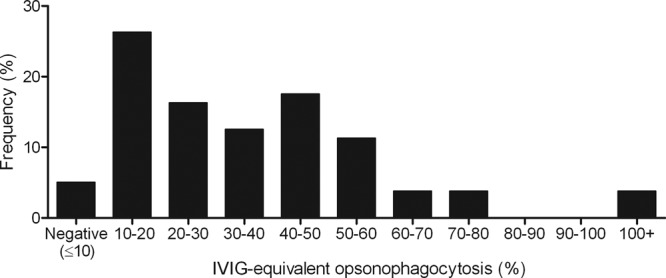
Immunity to *emm*1 GAS among healthy pregnant women. The ability of antenatal sera to opsonize FITC-labeled M1 GAS is shown for 80 representative sera (out of 199). The percentage of neutrophils associated with serum-opsonized FITC-labeled bacteria was measured by flow cytometry and expressed relative to the percentage of neutrophils associated with FITC-labeled bacteria when IVIG (2.5 mg/ml) was used as the opsonin. Negative, below the level of the “no serum” (complement alone) control (≤10% of IVIG-equivalent opsonophagocytosis).

The ability of antenatal sera to opsonize FITC-labeled *emm*1 GAS was determined by coincubation with fresh human neutrophils and measurement of subsequent phagocytosis; 5% of samples demonstrated no ability to facilitate opsonophagocytosis of *emm*1 GAS, while only 4% showed activity equivalent to that seen when we tested human IVIG (Fig. 3).

## DISCUSSION

This lethal outbreak of *emm*1 S. pyogenes in a maternity unit was explosive, commencing with a likely community source. Transmission events occurred on a single day with apparently transient contacts. Whole-genome sequencing confirmed cases to be clonal, although events were so rapid that a chronological sequence of transmission could be determined only by changes in a virulence gene, *sic*, rather than conventional analysis of whole-genome data.

The rapid and lethal nature of this outbreak highlights a number of learning points. First, GAS infection remains a deadly threat in the puerperium. The transmission risk within the maternity setting is high, not only to HCWs who attend infected patients, but also between social and family contacts and to the newborn infant. Oropharyngeal carriage may be important, and single episodes of contact are sufficient for productive transmission to occur (including both social and health care contact). Recognized risk factors for postpartum sepsis may not always be present, and signs of severe sepsis may be masked or present atypically. Invasive GAS infection in the postpartum period is not limited to classical genital tract (puerperal) sepsis and may present to physicians other than obstetricians, progressing rapidly to irreversible multiorgan failure.

Remarkably, sepsis is now the leading cause of “direct” maternal deaths in the United Kingdom. GAS accounted for 13 of 25 sepsis-related maternal deaths reported in 2006–2008 by the United Kingdom Centre for Maternal and Child Enquiries (CMACE), although the reasons for the increases are unclear (8, 9). Although pairs of GAS infection caused by the same *emm* type have been previously reported in maternity units, such events are rare and may be related to HCW carriage (1, 10, 11). The outbreak we describe was caused by *emm*1 GAS, the major lineage associated with invasive disease in Europe and the United States. Although GAS puerperal sepsis is most frequently associated with *emm*28, the mortality of GAS puerperal infection is disproportionately attributable to *emm*1 and *emm*3 S. pyogenes isolates (12). Environmental factors that influence GAS outbreaks have been well documented (seasonal variation, sharing rooms, hygiene, etc.) (13–16), but the host and the pathogen factors affecting transmission of S. pyogenes in this setting are unknown.

The affected women were admitted while in labor to the same unit on the same day. It is not possible to definitively determine whether infection was acquired outside the hospital and then transmitted sequentially through person-to-person contact within the unit or whether the maternity patients acquired their infection from a common source. However, one isolate (out of four) from case 1 had undergone allelic change in *sic* at an unknown time point, indicative of longer mucosal colonization. Changes in *sic* within individual patients carrying GAS mucosally have been reported previously, as have changes during epidemics within a population (5, 17), but these events may require prolonged carriage (>2 weeks). Indeed, experimental mucosal infection of mice failed to induce allelic change in *sic* over a 9-day period (data not shown), a result consistent with those of other studies (5, 6). Due to the high numbers of repeat regions within the *sic* gene, the mapping software used to analyze whole-genome sequencing cannot identify such changes, necessitating conventional sequencing. Although both women delivered within minutes of one another, symptomatic infection in case 2 arose 2 days later than in case 1. We speculate that the administration of prophylactic beta lactam antibiotics for ruptured membranes in case 2 may have delayed the onset of disease, although these antibiotics were insufficient to prevent GAS acquisition or colonization.

The routes of GAS transmission in the puerperium are hard to investigate, since samples are seldom obtained from women antenatally or from contacts, except where an outbreak occurs. GAS is rarely present in the genital tract antenatally (18–21); hence, screening or clearance regimens, antenatally or intrapartum, are unlikely to have an impact on postpartum GAS infection. The importance of the respiratory tract as a source of infection in recently pregnant women was established in the 1930s, and reiterated in the CMACE report (8, 18). GAS throat carriage among healthy adults in England is low, around 0.5 to 2% (22), consistent with the 1% carriage rate (of nonoutbreak strains) we detected in HCWs who were not direct contacts of the cases. In contrast, 4.3% of the HCWs identified as contacts were found to be carriers or infected with the outbreak strain, suggesting that it may be especially suited to spreading in this setting. Transmission of the outbreak strain to HCWs was, however, limited to those reporting close but single clinical contacts, and this raises the question as to whether mask wearing is advisable for HCWs treating GAS patients. Screening of HCWs was performed using throat or skin swabs and thus excluded those carrying GAS rectally or vaginally. Previous outbreaks have been associated with HCWs asymptomatically carrying GAS at all mucosal sites (11); therefore, we cannot fully exclude an unknown HCW source of GAS. HCW compliance is essential during outbreak investigation (13, 23), and newly issued United Kingdom guidance (14) recommends that nasal, rectal, and vaginal swabs should be obtained, if other swabs are negative, in situations where an HCW is implicated in transmission.

Contact with children in either the home or the workplace has been reported in association with maternal invasive GAS infection (8); coincidentally, both women who died were teachers, although they taught at separate schools. While it is possible that children represented a reservoir for the outbreak strain, the strain was not detected in any of 193 HCWs that were not contacts of the patients, nor was it identified among 100 GAS throat isolates submitted from the affected region in the 1 year following the outbreak. We therefore concluded that the outbreak strain was not circulating in the wider community.

Women after delivery are 20 times more susceptible to hemolytic streptococcal bacteremia than nonpregnant women; this risk increases to 100-fold if all invasive GAS infections are included (24, 25). There is a pressing need to establish a robust assay for GAS immunity that can be applied to large populations. To determine immunity in a similar but unrelated antenatal population, we used an assay that solely measured serum opsonic function that could result in phagocytosis. Five percent of antenatal sera showed no opsonic activity against *emm*1 GAS, highlighting an underlying susceptibility to GAS infection in a significant proportion of the antenatal population. Whether immunity to GAS alters during the course of pregnancy is entirely unknown and is a subject of ongoing research.

To our knowledge, the *emm*1 GAS isolates in this study are the first to be sequenced outside the United States. For comparison and SNP identification, we chose to align our sequences against the *emm*1 genome strain MGAS5005 (rather than SF370), as the MGAS5005 strain is more representative of the globally disseminated *emm*1 clone (3). Mutations in regulatory genes are reportedly characteristic of *emm*1 hyperinvasiveness (26–28) but may result in fitness cost, reducing colonization potential (29). These mutations were not observed in any of the outbreak strains and were observed in fewer than half of the invasive *emm*1 strains from elsewhere in England. Preservation of GAS regulatory gene function may have underpinned the rapid transmissibility and adaptability essential for this explosive but short-lived outbreak.

This outbreak illustrates the devastating rapidity and intensity with which GAS can spread within a susceptible population and draws attention to the need for urgent infection control intervention, including immediate staff screening in a suspected outbreak to prevent consequent additional transmission events. Staff screening should occur prior to the availability of the results of molecular typing and ideally before staff members return to duty, although this may necessitate a flexible approach to the screening process rather than the conventional use of occupational health units.

The *emm*1 GAS organism is equipped with a range of virulence factors that allow it to cause the spectrum of disease observed in this outbreak (30), from asymptomatic colonization through to tonsillitis, hemorrhagic pneumonia, and bacteremia toxic shock. Recent guidelines regarding outbreak prevention (14) and management of severe sepsis in the obstetric patient are timely (31). When reviewing maternity patients presenting after childbirth, vigilance is needed with regard to suspicion of sepsis, which may develop in the community, as occurred in both cases reported here.

## Supplementary Material

Supplemental material
