# Supplementary material for: Molecular Analysis of an Outbreak of Lethal Postpartum Sepsis Caused by Streptococcus pyogenes
Source: J Clin Microbiol. 2013 Jul;51(7):2089–95. doi: 10.1128/JCM.00679-13 (PMC3697669; doi:10.1128/JCM.00679-13)
Supplement: Supplemental material [file supp_51_7_2089__index.html]

Molecular Analysis of an Outbreak of Lethal Postpartum Sepsis Caused by Streptococcus pyogenes — Supplemental material 

# Molecular Analysis of an Outbreak of Lethal Postpartum Sepsis Caused by Streptococcus pyogenes

## 

**Files in this Data Supplement:**

- Supplemental file 1 -

  Supplemental methods; Figures S1 (Maternity unit isolates were phenotypically indistinguishable from other *emm*1 isolates), S2 (The outbreak *emm*1 GAS strains demonstrated the unique SIC alleles SIC1.300 and SIC1.301), S3 (Immunity to *sic* alleles among healthy pregnant women), and S4 (Immunoreactivity of IgG in antenatal sera against whole *emm*1 GAS cells, measured relative to standard concentrations of IVIG); and Tables S1 (*Streptococcus pyogenes* isolates used in this study), S2 (Single nucleotide polymorphisms identified in the core genomes of maternity unit isolates and other *emm*1 isolates), S3 (Insertions and deletions identified in the core genomes of maternity unit isolates), and S4 (Insertions and deletions identified in the core genomes of other *emm*1 isolates)

  PDF, 835K
