# Supplementary material for: Molecular Analysis of an Outbreak of Lethal Postpartum Sepsis Caused by Streptococcus pyogenes
Source: J Clin Microbiol. 2013 Jul;51(7):2089–95. doi: 10.1128/JCM.00679-13 (PMC3697669; doi:10.1128/JCM.00679-13)
Supplement: Supplemental material [file JCM.00679-13_zjm999092597so1.pdf]

Lethal *Streptococcus pyogenes* post-partum sepsis: Molecular analysis of an outbreak

Supplementary Appendix

Supplementary Methods

Bacterial strains

GAS strains were cultured in Todd Hewitt broth (Oxoid) or on Columbia blood agar plates (Oxoid) at 37°C, 5% CO_2. Penicillin minimal inhibitory concentrations were determined in liquid culture according to the protocol described by Andrews (1). *Escherichia coli* strains were cultured in Luria-Bertani broth (Oxoid).

Genotyping

GAS *emm* sequencing and sub-typing was performed according to the protocol of the Centers for Diseases Control and Prevention (www.cdc.gov/ncidod/biotech/strep/protocol_emm-type). Genotyping for superantigen genes was performed using multiplex polymerase chain reaction using the method previously reported (2) but analyzed by agarose gel electrophoresis. Amplification and sequencing of the *covR/S* locus and the *sic* gene was performed as previously described (3,4). Alleles of *sic* were determined by comparison to a reference *sic* allele (GenBank X92968) (5) and assigned allele numbers based on identity to available sequences. New *sic*-alleles were assigned sequential numbers and deposited on GenBank (Sic1.300; JF930695, Sic1.301; JF930696, Sic1.302; JF930697, Sic1.303; JF930698, Sic1.304; JF930699, Sic1.305; JF930700, Sic1.306; JF930701, Sic1.307; JF930702, Sic1.308; JF930703, Sic1.309; JF930704).

Phenotypic analysis

Streptococcal cysteine protease, SpeB was measured in stationary phase bacterial culture supernatant using western blot analysis probed with anti-SpeB antibody (Toxin Technology, Sarasota, USA). SpeB concentration was measured against a standard curve of recombinant SpeB (Toxin Technology) using densitometry. *S. pyogenes* cell envelope proteinase, SpyCEP and hyaluronic acid capsule was measured in mid-logarithmic phase of growth using methods described previously (6, 7). Resistance to opsonophagocytosis was measured in whole human blood as previously described (8). Briefly approximately 50 colony forming units (CFU) of GAS were used to inoculate 300µl of freshly extracted heparinized human blood. Cultures were incubated at 37°C for 3 hours rotating after which final CFU were measured by plating onto blood agar. Multiplication factor was calculated by dividing the final CFU by the initial inoculum. Mitogenicity, as a measure of superantigen production, was tested using a standard human monoclonal cell (MNCs) proliferation assay as previously described (9). Three separate donors were used.

GAS whole genome sequencing

Multiplex paired-end Illumina sequencing was performed on 24 strains in two batches using 108bp or 76bp reads. Using SSAHA software (10), reads were mapped to the complete genome sequence of USA *emm*1 strain MGAS5005 (11) and single polymorphisms (SNPs) were identified. SNPs identified in the core genome were used to generate a Maximal Likelihood Tree using RAxML. The sequences were deposited on the short read archive under the accession numbers ERX009526 and ERX010030. Details of all SNPs and InDels can be found in Tables S2 and S3.

Recombinant sic proteins

Three *sic* alleles, *sic*1.02, *sic*1.300 and *sic*1.301 were cloned and expressed in *E. coli* using the expression vector pET19b (Novagen, Nottingham, UK). Recombinant proteins were nickel-column purified according to the manufacturers' instructions (Novagen).

Complement inhibition assay

Sheep red blood cells were washed in complement fixation diluent (CFD) (Oxoid) and sensitized with rabbit antisheep red blood cell stromal antibody (Sigma) for 15 minutes rotating at 4°C. Recombinant SIC protein was serially diluted in CFD and incubated with 2% sensitized erythrocytes plus 1% normal human serum obtained from a healthy donor, at 37°C for 30 minutes. After centrifugation at 400xg for 5 minutes, absorbance of the supernatant was measured at 415nm.

ELISA-based assays

Human sera were from anonymized antenatal screening samples obtained at ICHT in 2009 in accordance with approval from the West London Research Ethics Committee (reference 06/Q0406/20). Reactivity of human antenatal sera towards recombinant SIC proteins was tested using an ELISA-based method. Using a 96 well plate, 48 wells were coated with 20µg/ml of recombinant SIC protein in carbonate buffer (pH9.6) at 4°C 16 hours, the remaining 48 wells were left uncoated. Plates were then washed and blocked in PBS-5% nonfat milk. Human antenatal sera samples were diluted 1:100 in PBS-5% nonfat milk and 50µl was applied to both recombinant SIC protein-coated wells and uncoated wells in duplicate. Bound IgG was detected with goat antihuman IgG-HRP at 1:60,000. In order to quantify SIC-reactive IgG in each serum sample, the activity of pooled human intravenous immunoglobulin (IVIG) against recombinant SIC protein was also measured using a series of

fixed concentrations of IVIG, diluted 1 in 2 from 100µg/ml, to generate a standard curve of IVIG-equivalent antiSIC IgG on each plate. Final IVIG-equivalent antiSIC IgG levels in each serum sample were calculated against the standard curve and the values of uncoated well were subtracted from coated wells. Serum samples were diluted further if required. This was repeated for all three recombinant SIC proteins and 199 serum samples.

To detect IgG reactivity of human antenatal sera samples towards whole M1 (strain H584) *S. pyogenes* cells, H584 was cultured overnight in THB, washed twice in PBS and resuspended to an optical density (600nm) 0.5. Bacterial suspensions were then heated to 85°C for 45 minutes, aliquoted and stored at -20°C. Plates were coated 100µl/well of bacterial suspension at 4°C 16 hours then washed and blocked in PBS-5% nonfat milk. Human antenatal serum samples were diluted 1:5000 in PBS-5% nonfat milk and 50µl was applied to the wells in duplicate. Bound IgG was detected with goat antihuman IgG-HRP at 1:60,000. IVIG-equivalent antiGAS IgG in each serum sample was quantified using an IVIG standard similar to the method used for antiSIC IgG except the top concentration used was 10µg/ml. Final antiGAS IgG concentrations of each sample were calculated after subtracting the mean plus 2 standard deviations of negative serum samples. Negative serum samples were generated by removing specific GAS antibodies through adsorption. Briefly six human serum samples were diluted 1 in 5000 and incubated with heated killed bacterial suspension at 4°C for 1 hour, rotating. Bacteria were then removed by centrifugation and the process was repeated. Adsorbed serum samples were then used in the assay as described.

Neutrophil extraction

Human neutrophils were extracted from fresh heparinized human blood. Blood was added to an equal volume of 3% dextran (MW≥100,000) and 0.9% NaCl solution. After 30 minutes separation, cells were pelleted from the top layer by centrifugation at 214xg for 10 minutes

and resuspended in 0.9% NaCl solution before layering onto Ficoll-Paque™ plus (GE Healthcare, UK). Following centrifugation at 700xg for 40 minutes the top layers were discarded and red blood cells removed by hypotonic lysis with 0.2% NaCl solution. Neutrophils were counted and adjusted to $2x10^6$ cells/ml.

Opsonophagocytosis

This was performed with with 80 (of 199) representative heat-inactivated antenatal patient sera. M1 GAS (H584) were cultured overnight and washed in PBS before resuspending in carbonate buffer pH9 with 0.02mg/ml FITC. After incubation for 30 minutes at 37^oC rotating, bacterial cells were washed twice in PBS and adjusted to A_{600nm}0.35. For each reaction, 100µl bacterial suspension was opsonised with 200µl test serum at 37^oC for 30 minutes before adding to $2x10^6$ neutrophils and 10% complement (rabbit serum, Merck Chemicals, UK). After 30 minutes incubation at 37^oC rotation, opsonophagocytosis was stopped with ice cold 0.9% saline 0.02% EDTA and FITC-labelled bacteria associated neutrophils were measured on the flow cytometer. Antenatal-serum-opsonized GAS were compared with IVIG (2.5mg/ml)-opsonized GAS, as this has been shown to provide optimum opsonophagocytosis. Non-opsonized GAS were used as a negative control.

Figures

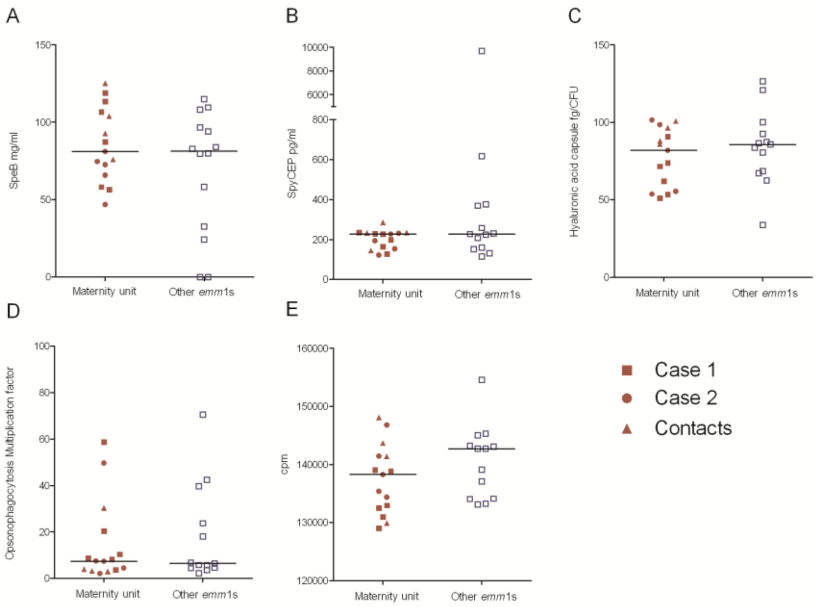

Figure S1. Maternity unit isolates were phenotypically indistinguishable from other *emm*1 isolates. All the strains associated with the outbreak were phenotypically tested including those isolated directly from maternity patient case 1 (red squares), and maternity patient case 2 (red circles) plus all isolates from household and healthcare worker contacts and additional maternity patient (red triangles). Other England circulating *emm*1 strains from the same time period were used as a comparison (n=13, blue squares). Although there was wide variation in the amount of the secreted cysteine protease, SpeB (A), chemokine cleaving *Streptococcus pyogenes* cell envelope proteinase, SpyCEP (B), and hyaluronic acid capsule (C) produced both by the maternity unit isolates and other *emm*1 strains, the two groups were indistinguishable from each other. Two *emm*1 strains produced undetectable amounts of SpeB and one *emm*1 strain produced large amounts of SpyCEP; these invasive strains were identified to have either a mutation in *rgg* or *covR/S*. Note that the isolate obtained from the additional maternity patient demonstrated no obvious deficiency in capsule expression despite a single T deletion from a poly-T-hexamer tract that generated a frameshift truncation mutation in one of the enzymes required to synthesise the capsule, HasB. Resistance to opsonophagocytosis (D), as measured by growth in whole human blood, also did not differ between the two groups. Neither was there an obvious difference in superantigen production (E), measured as counts per minute (cpm) of tritiated-thymidine uptake during a proliferative response of human mononuclear cells to streptococcal culture supernatant. Data are the mean of at least 2 experiments for each strain. Horizontal line; median.

A

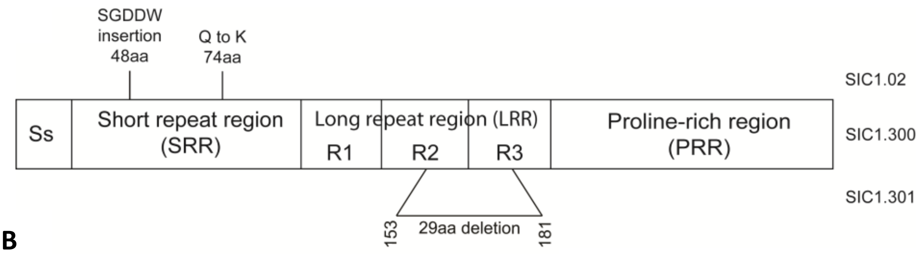

B

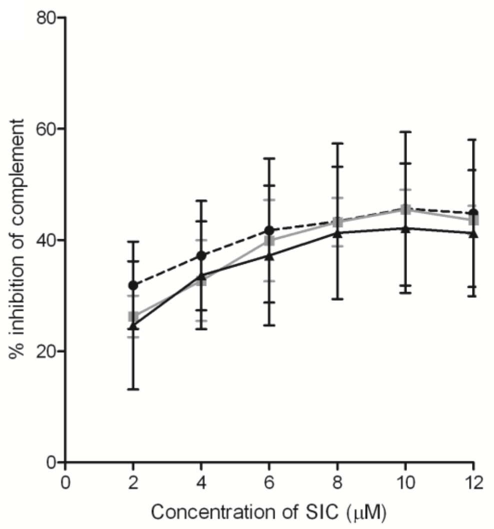

Figure S2. The outbreak *emm*1 GAS strains demonstrated the unique SIC alleles, SIC1.300 and SIC1.301. A. The domains of the SIC protein consist of a signal sequence (Ss), short repeat region (SRR), long repeat region (LRR) containing three long repeat regions, and the proline-rich region (PRR) [14]. SIC1.301 differed from SIC1.300 by a 29 amino acid deletion. The 29 amino acid (aa) deletion on SIC1.301 occurred within the long repeat region. SIC1.02 is the common SIC allele in the UK, and differs from both SIC1.300 and SIC1.301 by an insertion at 48aa and an amino acid substitution of glutamine (Q) to lysine (K). B. Inhibition of complement mediated lysis is not affected by differences in *sic* alleles. Sensitized sheep red blood cells were incubated with human sera, as a source of complement, and increasing amounts of each recombinant SIC protein; SIC1.300 (grey, squares), SIC1.301 (black, triangles) and SIC1.02 (dotted, circles). Data are mean (±standard deviation) of three separate experiments measured in duplicate.

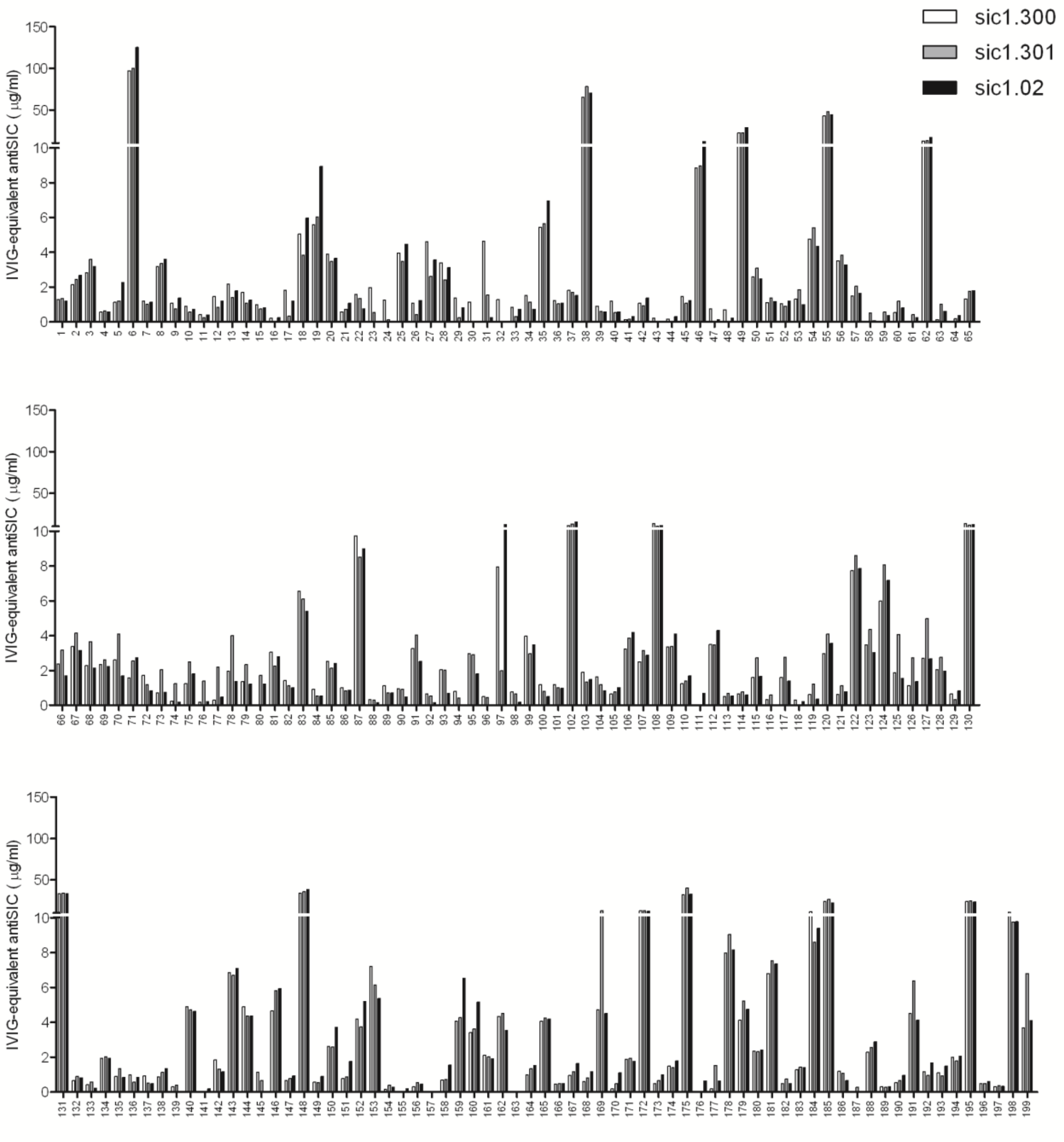

Figure S3. Immunity to *sic* alleles among healthy pregnant women. Immunoreactivity of IgG in antenatal sera against recombinant SIC proteins, measured relative to standard concentrations of pooled human intravenous immunoglobulin (IVIG) and expressed as μg/ml. All 199 sera tested shown individually. Three different SIC alleles were tested; SIC1.300 (white bars, associated with outbreak strains); SIC1.301 (grey bars, associated with one outbreak strain), and SIC1.02 (black bars, common type in UK, not associated with the outbreak strain).

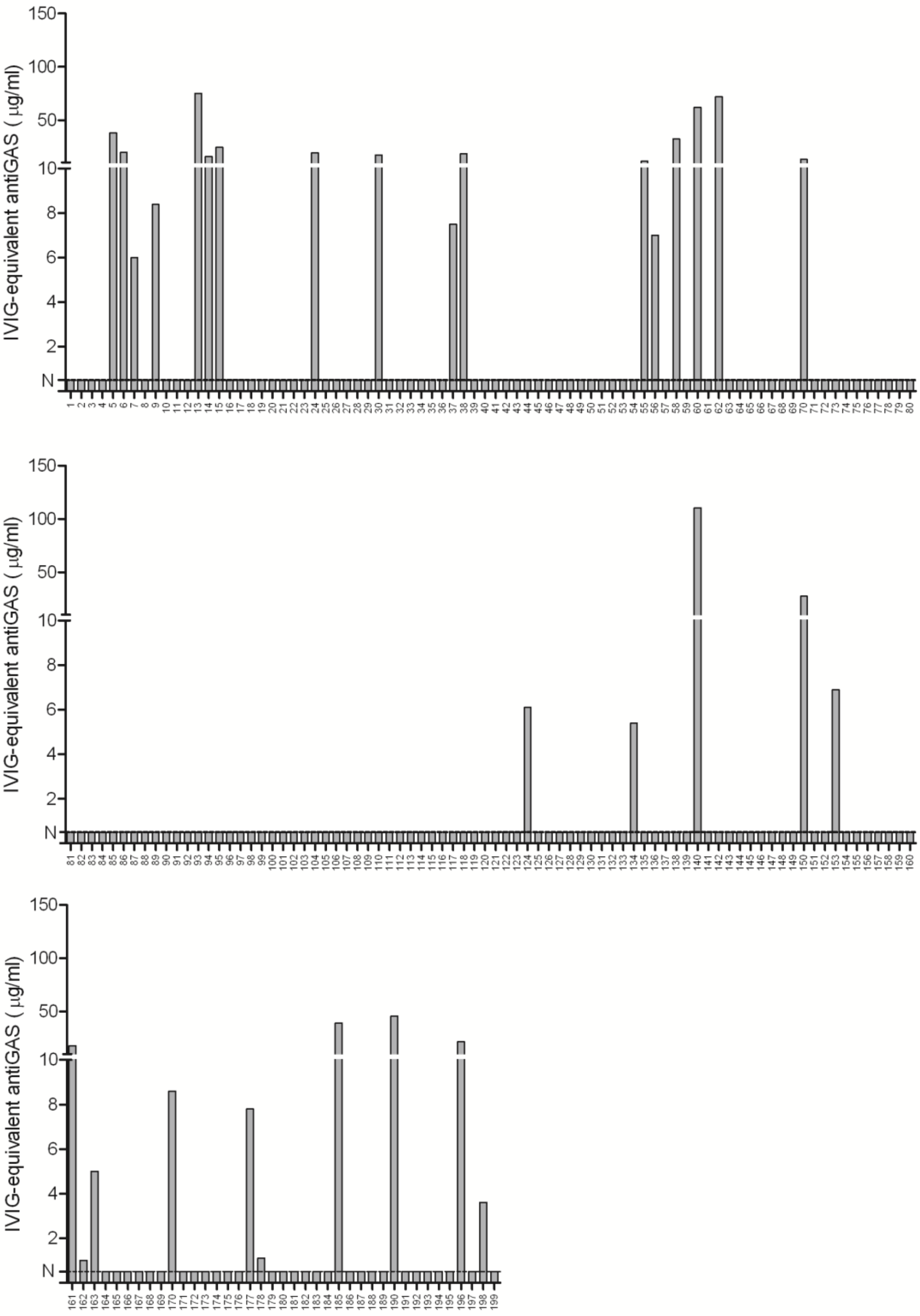

Figure S4. Immunoreactivity of IgG in antenatal sera against whole *emm*1 GAS cells, measured relative to standard concentrations of IVIG and expressed as µg/ml. All 199 sera tested shown individually. N; negative based on the level of non-specific reactivity.

Table S1. *Streptococcus pyogenes* isolates used in this study.

Isolate description		sic-type	Date isolated
**Outbreak cluster**			
Case 1	Maternity patient blood isolate	sic1.300	22/12/2007
	Maternity patient blood isolate	sic1.300	22/12/2007
	Maternity patient cervical isolate	sic1.300	24/12/2007
	Maternity patient genital tract (GT) isolate	sic1.301	23/12/2007
	Household contact nasopharyngeal isolate	sic1.300	24/12/2007
	Baby nasopharyngeal isolate	sic1.300	23/12/2007
Case 2	Maternity patient upper respiratory tract (URT) isolate (taken in A&E)	sic1.300	24/12/2007
	Maternity patient upper respiratory tract (URT) isolate	sic1.300	24/12/2007
	Maternity patient lower respiratory tract (LRT) isolate	sic1.300	27/12/2007
	Maternity patient lower respiratory tract (LRT) isolate	sic1.300	27/12/2007
	Household contact nasopharyngeal isolate	sic1.300	28/12/2007
Contacts	Doctor nasopharyngeal isolate	sic1.300	29/12/2007
	Midwife nasopharyngeal isolate	sic1.300	31/12/2007
	ICU nurse nasopharyngeal isolate	sic1.300	02/01/2008
	Maternity patient nasopharyngeal isolate	sic1.300	10/01/2008
**Other England *emm*1 isolates**			
Sequenced	Throat isolate	sic1.13	Jan 2008
	Blood isolate	sic1.13	Jan 2008
	Blood isolate	sic1.304	Dec 2007
	Blood isolate	sic1.02	Sept 2007
	Blood isolate	sic1.303	Sept 2007
	Blood isolate	sic1.02	Jan 2008
	Blood isolate	sic1.307	Dec 2007
	Blood isolate	sic1.306	Nov 2007
	Throat isolate*	sic1.309	Jul 2009
Additional	Pus isolate	sic1.02	Sept 2007
	Pleural fluid isolate	sic1.296	Nov 2007
	Post-mortem lung isolate	sic1.302	Oct 2007
	Blood isolate	sic1.305	Oct 2007
	Blood isolate	sic1.308	Nov 2007

*Isolated from the same area as the reference cases, not included in phenotype analysis.

Table S2. Single nucleotide polymorphisms identified in the core genomes of maternity unit isolates and other *emm*1 isolates.

							Case 1						Case 2					Contacts				Other *emm* 1 isolates								
**Locus**	**Gene**	**S/N**[*]	**Strand**	**Position**	**Ref base**	**SNP base**	BC[†]	BC	Cx[‡]	GT[¥]	HH[$] Contact	Baby	LRT[‖]	LRT	URT[¶]	URT	HH Contact	Doctor	Midwife	Nurse	Postpartum	Throat	Blood	Throat	Blood	Blood	Blood	Blood	Blood	Blood
M5005_Spy0010	-	N	1	9875	C	T	.	.	.	.	.	.	.	.	.	.	.	.	.	.	.	.	.	.	.	.	+	.	.	.
M5005_Spy0013	ftsH	N	1	14056	A	G	.	.	.	.	.	.	.	.	.	.	.	.	.	.	.	.	.	.	.	+	.	.	.	.
M5005_Spy0018	prsA.2	N	1	33536	G	A	+	+	+	+	+	+	+	+	+	+	+	+	+	+	+	+	+	+	+	+	.	.	.	+
M5005_Spy0023	-	N	1	38893	C	T	.	.	.	.	.	.	.	.	.	.	.	.	.	.	.	.	.	.	.	.	.	+	+	.
M5005_Spy0058	rpsH	N	1	71410	G	A	.	.	.	.	.	.	.	.	.	.	.	.	.	.	.	.	.	.	.	.	.	.	.	+
Intergenic	-	-	-	73744	C	T	.	.	.	.	.	.	.	.	.	.	.	.	.	.	.	.	.	.	.	.	+	.	.	.
M5005_Spy0069	rpsK	S	1	77389	T	G	.	.	.	.	.	.	.	.	.	.	.	.	.	.	.	.	.	.	.	.	+	.	.	.
M5005_Spy0070	rpoA	S	1	77755	A	G	.	.	.	.	.	.	.	.	.	.	.	.	.	.	.	.	.	.	.	.	.	+	+	.
M5005_Spy0084	rpoC	N	1	98480	G	A	.	.	.	.	.	.	.	.	.	.	.	.	.	.	.	.	.	+	.	.	.	.	.	.
M5005_Spy0084	rpoC	S	1	98983	C	T	.	.	.	.	.	.	.	.	.	.	.	.	.	.	.	.	.	.	.	.	+	.	.	.
M5005_Spy0084	rpoC	N	1	99970	G	C	.	.	.	.	.	.	.	.	.	.	.	.	.	.	.	.	.	.	.	.	+	.	.	.
M5005_Spy0086	comYA	N	1	102728	A	G	.	.	.	.	.	.	.	.	.	.	.	.	.	.	.	.	.	.	.	.	.	.	+	.
M5005_Spy0094	ackA	N	1	107662	C	T	.	.	.	.	.	.	.	.	.	.	.	.	.	.	.	.	.	.	+	.	.	.	.	.
M5005_Spy0101	-	S	1	111924	G	A	+	+	+	+	+	+	+	+	+	+	+	+	+	+	+	+	+	+	+	+	.	.	.	+
M5005_Spy0102	-	N	1	112353	C	T	.	.	.	.	.	.	.	.	.	.	.	.	.	.	.	.	.	.	.	+	.	.	.	.
M5005_Spy0112	-	S	-1	123488	A	G	.	.	.	.	.	.	.	.	.	.	.	.	.	.	.	.	.	.	.	+	.	.	.	.
M5005_Spy0119	-	N	1	130651	G	A	.	.	.	.	.	.	.	.	.	.	.	.	.	.	.	.	.	.	.	.	+	.	.	.
Intergenic	-	-	-	147855	G	T	.	.	.	.	.	.	.	.	.	.	.	.	.	.	.	.	+	.	.	.	.	.	.	.
M5005_Spy0142	-	N	1	154111	A	G	.	.	.	.	.	.	.	.	.	.	.	.	.	.	.	.	.	.	.	.	.	+	+	.
M5005_Spy0146	metB	S	1	156703	G	T	.	.	.	.	.	.	.	.	.	.	.	.	.	.	.	.	.	.	.	.	.	+	+	.
Intergenic	-	-	-	160498	C	T	.	.	.	.	.	.	.	.	.	.	.	.	.	.	.	.	.	+	.	.	.	.	.	.
M5005_Spy0150	-	N	1	162677	C	T	+	+	+	+	+	+	+	+	+	+	+	+	+	+	+	+	+	.	.	.	.	.	.	.
Intergenic	-	-	-	167458	C	T	+	+	+	+	+	+	+	+	+	+	+	+	+	+	+	+	+	+	+	+	.	.	.	+
M5005_Spy0159	polA	S	1	172940	C	T	.	.	.	.	.	.	.	.	.	.	.	.	.	.	.	.	.	.	.	.	.	+	+	.
M5005_Spy0159	polA	S	1	173936	T	C	.	.	.	.	.	.	.	.	.	.	.	.	.	.	.	.	.	.	.	.	.	+	+	.
M5005_Spy0161	perR	N	1	175503	G	A	.	.	.	.	.	.	.	.	.	.	.	.	.	.	.	.	.	+	.	.	.	.	.	.
M5005_Spy0188	-	S	1	197178	T	C	+	+	+	+	+	+	+	+	+	+	+	+	+	+	+	.	.	.	+	+	+	.	.	.
M5005_Spy0205	fasC	N	1	212267	A	G	.	.	.	.	.	.	.	.	.	.	.	.	.	.	.	.	.	.	.	.	.	.	.	+
M5005_Spy0216	-	S	1	222140	A	G	.	.	.	.	.	.	.	.	.	.	.	.	.	.	.	+	.	.	.	.	.	.	.	.
M5005_Spy0222	ksgA	N	1	227119	C	T	+	+	+	+	+	+	+	+	+	+	+	+	+	+	+	+	+	.	.	.	.	.	.	.
M5005_Spy0223	-	N	1	228873	G	A	.	.	.	.	.	.	.	.	.	.	.	.	.	.	.	.	.	.	.	.	.	.	+	.

							Case 1						Case 2					Contacts				Other *emm* 1 isolates								
**Locus**	**Gene**	**S/N**	**Strand**	**Position**	**Ref base**	**SNP base**	BC[‡]	BC	Cx[‡]	GT[¥]	HH[$] Contact	Baby	LRT[‖]	LRT	URT[¶]	URT	HH Contact	Doctor	Midwife	Nurse	Postpartum	Throat	Blood	Throat	Blood	Blood	Blood	Blood	Blood	Blood
M5005_Spy0231	rpsG	N	1	237142	C	T	+	+	+	+	+	+	+	+	+	+	+	+	+	+	+	+	+	+	+	+	+	+	+	+
M5005_Spy0231	rpsG	S	1	237143	T	A	+	+	+	+	+	+	+	+	+	+	+	+	+	+	+	+	+	+	+	+	+	+	+	+
M5005_Spy0232	fus	S	1	237893	A	G	+	+	+	+	+	+	+	+	+	+	+	+	+	+	+	+	+	+	+	+	+	+	+	+
M5005_Spy0233	fus	S	1	238094	A	G	+	+	+	+	+	+	+	+	+	+	+	+	+	+	+	+	+	+	+	+	+	+	+	+
M5005_Spy0237	-	N	1	244993	C	A	+	+	+	+	+	+	+	+	+	+	+	+	+	+	+	+	+	+	+	+	+	+	+	+
M5005_Spy0238	bacA	S	1	246873	G	A	.	.	.	.	.	.	.	.	.	.	.	.	.	.	.	.	.	+	.	.	.	.	.	.
M5005_Spy0247	-	N	-1	254882	A	C	+	+	+	+	+	+	+	+	+	+	+	+	+	+	+	+	+	+	+	+	+	+	+	+
M5005_Spy0248	dacA2	N	-1	256244	T	C	+	+	+	+	+	+	+	+	+	+	+	+	+	+	+	+	+	+	+	+	+	+	+	+
M5005_Spy0259	-	S	1	272574	T	C	+	+	+	+	+	+	+	+	+	+	+	+	+	+	+	.	.	.	.	.	.	.	.	.
M5005_Spy0274	braB	N	-1	284512	C	T	.	.	.	.	.	.	.	.	.	.	.	.	.	.	.	.	.	.	.	.	.	.	.	+
M5005_Spy0275	-	N	1	285932	T	C	.	.	.	.	.	.	.	.	.	.	.	.	.	.	.	.	.	.	+	.	.	.	.	.
M5005_Spy0281	-	N	1	291765	C	T	+	+	+	+	+	+	+	+	+	+	+	+	+	+	+	+	+	+	+	+	.	.	.	+
M5005_Spy0283	covS	N	1	293490	C	T	.	.	.	.	.	.	.	.	.	.	.	.	.	.	.	.	.	.	.	.	.	.	+	.
M5005_Spy0283	covS	N	1	293786	C	T	.	.	.	.	.	.	.	.	.	.	.	.	.	.	.	.	.	.	.	.	+	.	.	.
M5005_Spy0287	pgdA	S	1	297645	C	T	.	.	.	.	.	.	.	.	.	.	.	.	.	.	.	.	.	.	+	+	.	.	.	.
M5005_Spy0288	snf	N	1	300520	C	T	.	.	.	.	.	.	.	.	.	.	.	.	.	.	.	.	.	.	.	.	+	.	.	.
M5005_Spy0290	murC	N	1	304244	T	C	.	.	.	.	.	.	.	.	.	.	.	.	.	.	.	.	.	+	.	.	.	.	.	.
M5005_Spy0307	-	N	1	316170	C	A	.	.	.	.	.	.	.	.	.	.	.	.	.	.	.	.	.	.	.	.	.	.	.	+
M5005_Spy0308	-	S	1	317175	A	G	.	.	.	.	.	.	.	.	.	.	.	.	.	.	.	.	.	.	.	.	.	.	.	+
M5005_Spy0331	dnaX	N	1	337731	C	T	+	+	+	+	+	+	+	+	+	+	+	+	+	+	+	+	+	.	.	.	.	.	.	.
M5005_Spy0331	dnaX	N	1	338178	A	G	+	+	+	+	+	+	+	+	+	+	+	+	+	+	+	+	+	+	+	+	+	+	+	+
Intergenic	-	-	-	343224	C	T	.	.	.	.	.	.	.	.	.	.	.	.	.	.	.	.	.	.	.	.	+	.	.	.
Intergenic	-	-	-	351241	G	T	.	.	.	.	.	.	.	.	.	.	.	.	.	.	.	.	.	.	+	.	.	.	.	.
Intergenic	-	-	-	358610	A	G	.	.	.	.	.	.	.	.	.	.	.	.	.	.	.	.	.	+	.	.	.	.	.	.
M5005_Spy0365	pfs	N	1	370653	C	T	.	.	.	.	.	.	.	.	.	.	.	.	.	.	.	.	.	.	+	.	.	.	.	.
M5005_Spy0377	pyrH	N	1	381937	A	G	+	+	+	+	+	+	+	+	+	+	+	+	+	+	+	+	+	+	+	+	+	+	+	+
M5005_Spy0377	pyrH	N	1	381938	T	C	+	+	+	+	+	+	+	+	+	+	+	+	+	+	+	+	+	+	+	+	+	+	+	+
M5005_Spy0377	pyrH	N	1	382109	A	C	+	+	+	+	+	+	+	+	+	+	+	+	+	+	+	+	+	+	+	+	+	+	+	+
Intergenic	-	-	-	384053	C	T	.	.	.	.	.	.	.	.	.	.	.	.	.	.	.	.	.	.	.	.	.	.	+	.
M5005_Spy0410	-	N	1	403151	C	T	.	.	.	.	.	.	.	.	.	.	.	.	.	.	.	.	.	.	.	.	+	.	.	.
M5005_Spy0419	-	S	-1	411165	A	C	+	+	+	+	+	+	+	+	+	+	+	+	+	+	+	+	+	.	.	.	.	.	.	.

							Case 1						Case 2					Contacts				Other *emm* 1 isolates								
**Locus**	**Gene**	**S/N**	**Strand**	**Position**	**Ref base**	**SNP base**	BC†	BC	Cx‡	GT¥	HH$ Contact	Baby	LRT‖	LRT	URT¶	URT	HH Contact	Doctor	Midwife	Nurse	Postpartum	Throat	Blood	Throat	Blood	Blood	Blood	Blood	Blood	Blood
Intergenic	-	-	-	444330	C	T	.	.	.	.	.	.	.	.	.	.	.	.	.	.	.	.	.	.	.	.	.	+	+	.
M5005_Spy0455	-	N	-1	449338	G	A	.	.	.	.	.	.	.	.	.	.	.	.	.	.	.	.	.	.	.	+	.	.	.	.
M5005_Spy0467	-	N	-1	458340	C	T	+	+	+	+	+	+	+	+	+	+	+	+	+	+	+	+	+	.	.	.	.	.	.	.
M5005_Spy0472	ftsY	N	1	461194	C	T	.	.	.	.	.	.	.	.	.	.	.	.	.	.	.	.	.	.	.	.	.	.	.	+
M5005_Spy0475	-	S	1	466103	G	A	.	.	.	.	.	.	.	.	.	.	.	.	.	.	.	.	.	+	.	.	.	.	.	.
M5005_Spy0484	ptsK	S	1	473934	T	C	+	+	+	+	+	+	+	+	+	+	+	+	+	+	+	+	+	+	+	+	+	+	+	+
Intergenic	-	-	-	481514	A	G	+	+	+	+	+	+	+	+	+	+	+	+	+	+	+	.	.	.	.	.	.	.	.	.
M5005_Spy0496	-	N	1	482472	G	A	.	.	.	.	.	.	.	.	.	.	.	.	.	.	.	.	.	.	+	.	.	.	.	.
M5005_Spy0516	pacL	S	1	502927	G	A	.	.	.	.	.	.	.	.	.	.	.	.	.	.	.	.	.	+	.	.	.	.	.	.
M5005_Spy0522	-	N	-1	510306	A	C	.	.	.	.	.	.	.	.	.	.	.	.	.	.	.	.	.	.	.	.	+	.	.	.
M5005_Spy0524	idnO	N	1	512389	C	A	.	.	.	.	.	.	.	.	.	.	.	.	.	.	.	.	.	.	.	+	.	.	.	.
M5005_Spy0535	-	N	1	522570	C	A	.	.	.	.	.	.	.	.	.	.	.	.	.	.	.	.	.	.	.	+	.	.	.	.
M5005_Spy0537	aspC	N	1	526076	G	A	.	.	.	.	.	.	.	.	.	.	.	.	.	.	.	.	.	.	.	.	+	.	.	.
M5005_Spy0547	-	S	-1	537572	C	T	+	+	+	+	+	+	+	+	+	+	+	+	+	+	+	+	+	.	.	.	.	.	.	.
M5005_Spy0547	-	S	-1	538085	T	C	.	.	.	.	.	.	.	.	.	.	.	.	.	.	.	.	.	.	.	.	+	.	.	.
M5005_Spy0553	gyrB	S	1	544617	C	T	.	.	.	.	.	.	.	.	.	.	.	.	.	.	.	.	.	.	.	.	+	.	.	.
M5005_Spy0556	eno	N	1	547723	C	G	+	+	+	+	+	+	+	+	+	+	+	+	+	+	+	+	+	+	+	+	+	+	+	+
M5005_Spy0556	eno	N	1	547727	T	A	+	+	+	+	+	+	+	+	+	+	+	+	+	+	+	+	+	+	+	+	+	+	+	+
M5005_Spy0556	eno	N	1	547733	G	T	+	+	+	+	+	+	+	+	+	+	+	+	+	+	+	+	+	+	+	+	+	+	+	+
M5005_Spy0556	eno	N	1	547738	C	T	+	+	+	+	+	+	+	+	+	+	+	+	+	+	+	+	+	+	+	+	+	+	+	+
M5005_Spy0556	eno	N	1	548633	G	T	+	+	+	+	+	+	+	+	+	+	+	+	+	+	+	+	+	.	.	.	.	.	.	.
Intergenic	-	-	-	559285	G	A	.	.	.	.	.	.	.	.	.	.	.	.	.	.	.	.	.	.	+	.	.	.	.	.
M5005_Spy0562	sagA	S	1	559807	A	G	+	+	+	+	+	+	+	+	+	+	+	+	+	+	+	+	+	+	+	+	.	.	.	+
M5005_Spy0578	atpH	N	1	577199	C	T	+	+	+	+	+	+	+	+	+	+	+	+	+	+	+	+	+	.	.	.	.	.	.	.
M5005_Spy0594	rexB	N	1	592918	A	C	+	+	+	+	+	+	+	+	+	+	+	+	+	+	+	+	+	+	+	+	+	+	+	+
Intergenic	-	-	-	600634	C	T	.	.	.	.	.	.	.	.	.	.	.	.	.	.	.	.	.	.	+	.	.	.	.	.
M5005_Spy0600	rpoD	N	1	603457	G	A	+	+	+	+	+	+	+	+	+	+	+	+	+	+	+	+	+	+	+	+	.	.	.	+
M5005_Spy0609	-	N	1	614327	C	T	.	.	.	.	.	.	.	.	.	.	.	.	.	.	.	.	.	.	.	.	+	.	.	.
M5005_Spy0609	-	N	1	614820	T	C	.	.	.	.	.	.	.	.	.	.	.	.	.	.	.	.	.	+	.	.	.	.	.	.
Intergenic	-	-	-	652131	T	G	+	+	+	+	+	+	+	+	+	+	+	+	+	+	+	.	.	.	.	.	.	.	.	.
Intergenic	-	-	-	652296	C	T	.	.	.	.	.	.	.	.	.	.	.	.	.	.	.	.	.	.	.	.	.	.	+	.

							Case 1						Case 2					Contacts				Other *emm* 1 isolates								
**Locus**	**Gene**	**S/N**	**Strand**	**Position**	**Ref base**	**SNP base**	BC[†]	BC	Cx[‡]	GT[¥]	HH[$] Contact	Baby	LRT[‖]	LRT	URT[¶]	URT	HH Contact	Doctor	Midwife	Nurse	Postpartum	Throat	Blood	Throat	Blood	Blood	Blood	Blood	Blood	Blood
M5005_Spy0647	-	S	1	653405	T	C	+	+	+	+	+	+	+	+	+	+	+	+	+	+	+	+	+	+	+	+	+	+	+	+
M5005_Spy0674	-	S	1	677694	C	T	.	.	.	.	.	.	.	.	.	.	.	.	.	.	.	.	.	.	.	.	.	+	+	.
M5005_Spy0684	mvaK2	N	1	687193	C	A	+	+	+	+	+	+	+	+	+	+	+	+	+	+	+	+	+	+	+	+	.	.	.	+
Intergenic	-	-	-	692168	A	G	.	.	.	.	.	.	.	.	.	.	.	.	.	.	.	.	.	.	.	.	+	.	.	.
M5005_Spy0699	deoD2	N	1	702761	A	C	.	.	.	.	.	.	.	.	.	.	.	.	.	.	.	.	.	.	.	.	.	.	.	+
M5005_Spy0727	recJ	S	1	731273	G	A	.	.	.	.	.	.	.	.	.	.	.	.	.	.	.	.	.	.	+	.	.	.	.	.
Intergenic	-	-	-	739670	T	A	.	.	.	.	.	.	.	.	.	.	.	.	.	.	.	.	.	.	.	.	.	+	+	.
M5005_Spy0757	hylA	S	-1	759533	T	C	.	.	.	.	.	.	.	.	.	.	.	.	.	.	.	.	.	.	.	.	.	+	+	.
M5005_Spy0763	femD	N	1	768317	C	A	+	+	+	+	+	+	+	+	+	+	+	+	+	+	+	+	+	+	+	+	+	+	+	+
M5005_Spy0764	-	N	1	769426	G	A	.	.	.	.	.	.	.	.	.	.	.	.	.	.	.	.	.	.	.	.	+	.	.	.
M5005_Spy0769	-	S	1	774179	G	A	.	.	.	.	.	.	.	.	.	.	.	.	.	.	.	.	.	.	.	+	.	.	.	.
M5005_Spy0774	-	N	1	779866	T	C	+	+	+	+	+	+	+	+	+	+	+	+	+	+	+	+	+	+	+	+	.	.	.	+
Intergenic	-	-	-	782222	A	G	+	+	+	+	+	+	+	+	+	+	+	+	+	+	+	+	+	+	+	+	+	+	+	+
M5005_Spy0786	-	N	1	791409	T	A	+	+	+	+	+	+	+	+	+	+	+	+	+	+	+	+	+	+	+	+	+	+	+	+
M5005_Spy0793	-	S	1	799040	T	C	.	.	.	.	.	.	.	.	.	.	.	.	.	.	.	.	.	.	.	.	.	+	+	.
M5005_Spy0812	-	S	-1	810947	G	A	.	.	.	.	.	.	.	.	.	.	.	.	.	.	.	.	.	.	.	.	.	+	+	.
M5005_Spy0839	-	N	1	833750	G	A	.	.	.	.	.	.	.	.	.	.	.	.	.	.	.	.	.	.	.	.	.	+	+	.
M5005_Spy0842	-	N	-1	836171	C	T	.	.	.	.	.	.	.	.	.	.	.	.	.	.	.	.	.	.	.	+	.	.	.	.
M5005_Spy0859	-	N	1	850210	A	T	+	+	+	+	+	+	+	+	+	+	+	+	+	+	+	+	+	+	+	+	+	+	+	+
M5005_Spy0861	-	S	-1	852265	A	G	.	.	.	.	.	.	.	.	.	.	.	.	.	.	.	.	.	+	.	.	.	.	.	.
M5005_Spy0862	tdk2	N	1	852864	C	T	.	.	.	.	.	.	.	.	.	.	.	.	.	.	.	.	.	.	+	+	.	.	.	.
M5005_Spy0882	-	N	1	872683	C	T	.	.	.	.	.	.	.	.	.	.	.	.	.	.	.	.	.	.	.	.	.	+	+	.
M5005_Spy0901	-	N	1	890169	C	T	.	.	.	.	.	.	.	.	.	.	.	.	.	.	.	.	.	.	.	.	.	+	+	.
M5005_Spy0903	oadB	N	1	891587	G	C	.	.	.	.	.	.	.	.	.	.	.	.	.	.	.	.	.	.	.	+	.	.	.	.
M5005_Spy0910	citC	N	-1	896926	T	A	.	.	.	.	.	.	.	.	.	.	.	.	.	.	.	.	.	.	.	.	+	.	.	.
M5005_Spy0935	dpfB	N	1	924764	G	A	.	.	.	.	.	.	.	.	.	.	.	.	.	.	.	.	.	.	.	.	+	.	.	.
M5005_Spy0938	pgmA	S	1	926451	G	A	.	.	.	.	.	.	.	.	.	.	.	.	.	.	.	.	.	.	.	.	.	+	.	.
M5005_Spy0938	pgmA	N	1	927358	G	A	.	.	.	.	.	.	.	.	.	.	.	.	.	.	.	.	.	.	.	.	.	+	+	.
M5005_Spy0947	ciaH	S	-1	937338	T	C	+	+	+	+	+	+	+	+	+	+	+	+	+	+	+	+	+	+	+	+	+	.	.	+
M5005_Spy0949	pepN	N	-1	940305	T	C	.	.	.	.	.	.	.	.	.	.	.	.	.	.	.	.	.	.	+	+	.	.	.	.
M5005_Spy0950	phoU	N	-1	942128	G	A	+	+	+	+	+	+	+	+	+	+	+	+	+	+	+	+	+	+	.	.	.	.	.	+

Locus	Gene	S/N	Strand	Position	Ref base	SNP base	Case 1						Case 2					Contacts				Other *emm* 1 isolates								
							BC†	BC	Cx‡	GT¥	HH$ Contact	Baby	LRT‖	LRT	URT¶	URT	HH Contact	Doctor	Midwife	Nurse	Postpartum	Throat	Blood	Throat	Blood	Blood	Blood	Blood	Blood	Blood
M5005_Spy0961	truB	N	-1	950766	C	T	.	.	.	.	.	.	.	.	.	.	.	.	.	.	.	.	.	+	.	.	.	.	.	.
M5005_Spy0963	-	N	-1	953599	A	G	.	.	.	.	.	.	.	.	.	.	.	.	.	.	.	.	.	.	.	.	.	.	+	.
M5005_Spy0965	-	N	-1	954841	T	C	.	.	.	.	.	.	.	.	.	.	.	.	.	.	.	.	.	+	.	.	.	.	.	.
Intergenic	-	-	-	957851	T	C	+	+	+	+	+	+	+	+	+	+	+	+	+	+	+	+	+	+	+	+	+	+	+	+
M5005_Spy0968	-	N	1	958230	A	C	.	.	.	.	.	.	.	.	.	.	.	.	.	.	.	.	.	.	.	.	+	.	.	.
M5005_Spy0968	-	N	1	958268	A	G	.	.	.	.	.	.	.	.	.	.	.	.	.	.	.	.	.	.	.	.	+	.	.	.
M5005_Spy0970	-	N	-1	959214	A	T	.	.	.	.	.	.	.	.	.	.	.	.	.	.	.	.	.	.	.	.	.	+	+	.
Intergenic	-	-	-	959311	C	T	+	+	+	+	+	+	+	+	+	+	+	+	+	+	+	+	+	+	+	+	.	.	.	+
Intergenic	-	-	-	966473	G	C	.	.	.	.	.	.	.	.	.	.	.	.	.	.	.	.	.	.	.	.	+	.	.	.
M5005_Spy0980	-	N	1	966656	G	A	.	.	.	.	.	.	.	.	.	.	.	.	.	.	.	.	.	.	.	.	.	.	+	.
M5005_Spy0984	-	S	-1	971041	T	C	.	.	.	.	.	.	.	.	.	.	.	.	.	.	.	.	.	.	.	.	.	+	+	.
M5005_Spy0988	pyk	S	-1	975747	G	A	.	.	.	.	.	.	.	.	.	.	.	.	.	.	.	.	.	.	.	.	.	.	+	.
M5005_Spy1055	glgP	N	-1	1023118	T	C	.	.	.	.	.	.	.	.	.	.	.	.	.	.	.	.	.	+	.	.	.	.	.	.
M5005_Spy1065	amyA	S	-1	1036924	A	G	.	.	.	.	.	.	.	.	.	.	.	.	.	.	.	.	.	+	.	.	.	.	.	.
Intergenic	-	-	-	1038101	C	T	.	.	.	.	.	.	.	.	.	.	.	.	.	.	.	.	.	.	.	.	.	+	+	.
M5005_Spy1066	amyB	S	-1	1039802	A	G	.	.	.	.	.	.	.	.	.	.	.	.	.	.	.	.	.	.	.	.	.	+	+	.
M5005_Spy1073	dltA	N	-1	1046960	T	C	+	+	+	+	+	+	+	+	+	+	+	+	+	+	+	+	+	+	+	+	+	+	+	+
M5005_Spy1076	glnH	N	1	1051858	G	A	.	.	.	.	.	.	.	.	.	.	.	.	.	.	.	.	.	.	+	.	.	.	.	.
M5005_Spy1085	bglA.2	N	-1	1059443	A	T	.	.	.	.	.	.	.	.	.	.	.	.	.	.	.	.	.	.	.	.	.	+	.	.
M5005_Spy1092	rsuA	S	-1	1064556	C	T	+	+	+	+	+	+	+	+	+	+	+	+	+	+	+	+	+	+	+	+	.	.	.	+
M5005_Spy1101	-	N	-1	1074902	C	T	.	.	.	.	.	.	.	.	.	.	.	.	.	.	.	.	.	.	.	.	+	.	.	.
M5005_Spy1109	inlA	N	-1	1085618	T	C	.	.	.	.	.	.	.	.	.	.	.	.	.	.	.	.	.	.	.	+	.	.	.	.
Intergenic	-	-	-	1085816	G	A	+	+	+	+	+	+	+	+	+	+	+	+	+	+	+	+	+	.	.	.	.	.	.	.
M5005_Spy1116	udk	N	-1	1091214	G	A	.	.	.	.	.	.	.	.	.	.	.	.	.	.	.	.	.	.	.	.	+	.	.	.
M5005_Spy1118	-	N	-1	1092987	T	C	.	.	.	.	.	.	.	.	.	.	.	.	.	.	.	.	.	.	.	.	.	+	+	.
M5005_Spy1120	pstI	S	-1	1096668	G	T	.	.	.	.	.	.	.	.	.	.	.	.	.	.	.	.	+	.	.	.	.	.	.	.
M5005_Spy1132	alaS	S	-1	1106894	C	T	.	.	.	.	.	.	.	.	.	.	.	.	.	.	.	.	.	.	.	.	.	.	.	+
M5005_Spy1133	surA	S	-1	1110340																							+			
M5005_Spy1144	-	N	-1	1120987	T	C	+	+	+	+	+	+	+	+	+	+	+	+	+	+	+	+	+	+	+	+	.	.	.	+
M5005_Spy1162	-	S	-1	1139774	A	G	+	+	+	+	+	+	+	+	+	+	+	+	+	+	+	+	+	+	+	+	+	+	+	+
M5005_Spy1162	-	N	-1	1139808	G	A	+	+	+	+	+	+	+	+	+	+	+	+	+	+	+	+	+	+	+	+	+	+	+	+

							Case 1						Case 2					Contacts				Other *emm*1 isolates								
**Locus**	**Gene**	**S/N**	**Strand**	**Position**	**Ref base**	**SNP base**	BC‡	BC	Cx‡	GT¥	HH$ Contact	Baby	LRT‖	LRT	URT¶	URT	HH Contact	Doctor	Midwife	Nurse	Postpartum	Throat	Blood	Throat	Blood	Blood	Blood	Blood	Blood	Blood
M5005_Spy1162	-	N	-1	1139985	G	A	+	+	+	+	+	+	+	+	+	+	+	+	+	+	+	+	+	+	+	+	+	+	+	+
M5005_Spy1162	-	S	-1	1140020	C	T	+	+	+	+	+	+	+	+	+	+	+	+	+	+	+	+	+	+	+	+	+	+	+	+
M5005_Spy1163	-	S	-1	1140903	T	C	.	.	.	.	.	.	.	.	.	.	.	.	.	.	.	.	.	.	+	.	.	.	.	.
M5005_Spy1226	-	N	-1	1181051	C	T	.	.	.	.	.	.	.	.	.	.	.	.	.	.	.	.	.	.	.	.	.	+	+	.
M5005_Spy1240	clpE	N	1	1194996	T	C	+	+	+	+	+	+	+	+	+	+	+	+	+	+	+	+	+	+	+	+	+	+	+	+
M5005_Spy1255	typA	N	-1	1212064	G	A	+	+	+	+	+	+	+	+	+	+	+	+	+	+	+	+	+	+	+	+	.	.	.	+
M5005_Spy1257	glcK	N	-1	1215153	T	C	.	.	.	.	.	.	.	.	.	.	.	.	.	.	.	.	.	.	.	.	+	.	.	.
M5005_Spy1258	-	N	-1	1215371	C	T	.	.	.	.	.	.	.	.	.	.	.	.	.	.	.	.	.	.	.	.	+	.	.	.
M5005_Spy1264	-	N	-1	1219753	T	C	+	+	+	+	+	+	+	+	+	+	+	+	+	+	+	+	+	+	+	+	+	+	+	+
Intergenic	-	-	-	1231069	G	A	.	.	.	.	.	.	.	.	.	.	.	.	.	.	.	.	.	.	.	.	.	+	+	.
M5005_Spy1279	-	N	-1	1233517	A	G	+	+	+	+	+	+	+	+	+	+	+	+	+	+	+	+	+	+	+	+	+	+	+	+
M5005_Spy1282	msrA	N	-1	1237632	G	C	.	.	.	.	.	.	.	.	.	.	.	.	.	.	.	.	.	+	.	.	.	.	.	.
M5005_Spy1286	-	N	-1	1241339	G	A	.	.	.	.	.	.	.	.	.	.	.	.	.	.	.	.	.	+	.	.	.	.	.	.
M5005_Spy1291	-	N	-1	1246590	G	A	+	+	+	+	+	+	+	+	+	+	+	+	+	+	+	+	+	.	.	.	.	.	.	.
M5005_Spy1291	-	N	-1	1247794	A	C	.	.	.	.	.	.	.	.	.	.	.	.	.	.	.	.	.	.	+	.	.	.	.	.
Intergenic	-	-	-	1251873	A	C	+	+	+	+	+	+	+	+	+	+	+	+	+	+	+	+	+	.	.	.	.	.	.	.
M5005_Spy1317	-	S	1	1277300	C	T	.	.	.	.	.	.	.	.	.	.	.	.	.	.	.	.	.	.	.	.	+	.	.	.
M5005_Spy1320	recX	S	1	1283161	C	T	.	.	.	.	.	.	.	.	.	.	.	.	.	.	.	.	.	+	.	.	.	.	.	.
M5005_Spy1327	comFA	S	-1	1293361	T	C	.	.	.	.	.	.	.	.	.	.	.	.	.	.	.	.	.	.	.	.	.	.	.	+
M5005_Spy1331	-	N	-1	1297598	G	A	.	.	.	.	.	.	.	.	.	.	.	.	.	.	.	.	.	.	.	.	+	.	.	.
M5005_Spy1377	-	S	-1	1349188	G	A	.	.	.	.	.	.	.	.	.	.	.	.	.	.	.	.	.	.	.	.	+	.	.	.
M5005_Spy1380	glpO	N	-1	1351675	A	G	.	.	.	.	.	.	.	.	.	.	.	.	.	.	.	.	+	.	.	.	.	.	.	.
M5005_Spy1381	glpK	S	-1	1354422	C	T	+	+	+	+	+	+	+	+	+	+	+	+	+	+	+	.	.	.	.	.	.	.	.	.
M5005_Spy1387	-	N	-1	1360756	G	A	.	.	.	.	.	.	.	.	.	.	.	.	.	.	.	.	.	.	.	.	.	+	+	.
M5005_Spy1401	-	N	-1	1373672	A	C	.	.	.	.	.	.	.	.	.	.	.	.	.	.	.	.	.	.	.	.	+	.	.	.
M5005_Spy1405	copA	S	-1	1376376	T	G	.	.	.	.	.	.	.	.	.	.	.	.	.	.	.	.	.	+	.	.	.	.	.	.
M5005_Spy1407	-	S	1	1378864	T	C	.	.	.	.	.	.	.	.	.	.	.	.	.	.	.	.	.	.	.	.	.	.	.	+
M5005_Spy1481	manN	S	1	1437924	C	A	.	.	.	.	.	.	.	.	.	.	.	.	.	.	.	.	.	.	.	.	+	.	.	.
Intergenic	-	-	-	1451610	C	A	.	.	.	.	.	.	.	.	.	.	.	.	.	.	.	.	.	.	+	.	.	.	.	.
M5005_Spy1500	hrcA	S	-1	1456608	A	G	.	.	.	.	.	.	.	.	.	.	.	.	.	.	.	.	.	+	.	.	.	.	.	.
M5005_Spy1503	-	N	-1	1458312	A	G	+	+	+	+	+	+	+	+	+	+	+	+	+	+	+	+	+	+	+	+	+	+	+	+

							Case 1						Case 2					Contacts				Other *emm* 1 isolates								
**Locus**	**Gene**	**S/N**	**Strand**	**Position**	**Ref base**	**SNP base**	BC†	BC	Cx‡	GT¥	HH$ Contact	Baby	LRT‖	LRT	URT¶	URT	HH Contact	Doctor	Midwife	Nurse	Postpartum	Throat	Blood	Throat	Blood	Blood	Blood	Blood	Blood	Blood
M5005_Spy1511	-	N	-1	1464247	A	G	+	+	+	+	+	+	+	+	+	+	+	+	+	+	+	+	+	+	+	+	+	+	+	+
M5005_Spy1514	-	N	1	1467368	T	C	+	+	+	+	+	+	+	+	+	+	+	+	+	+	+	+	+	+	+	+	+	+	+	+
M5005_Spy1514	-	S	1	1467480	G	A	.	.	.	.	.	.	.	.	.	.	.	.	.	.	.	.	.	.	+	.	.	.	.	.
M5005_Spy1525	-	N	-1	1480163	T	A	.	.	.	.	.	.	.	.	.	.	.	.	.	.	.	.	.	.	.	+	.	.	.	.
M5005_Spy1525	-	N	-1	1480693	T	C	.	.	.	.	.	.	.	.	.	.	.	.	.	.	.	.	.	+	.	.	.	.	.	.
M5005_Spy1530	-	N	-1	1487337	C	A	.	.	.	.	.	.	.	.	.	.	.	.	.	.	.	.	.	.	.	.	.	+	+	.
M5005_Spy1530	-	S	-1	1488154	A	G	+	+	+	+	+	+	+	+	+	+	+	+	+	+	+	+	+	+	+	+	+	+	+	+
M5005_Spy1537	-	N	-1	1495895	G	A	.	.	.	.	.	.	.	.	.	.	.	.	.	.	.	.	.	.	.	+	.	.	.	.
M5005_Spy1537	-	S	-1	1495896	A	G	.	.	.	.	.	.	.	.	.	.	.	.	.	.	.	.	.	.	.	+	.	.	.	.
M5005_Spy1542	scrA	N	-1	1502416	A	C	.	.	.	.	.	.	.	.	.	.	.	.	.	.	.	.	.	.	.	.	.	.	.	+
Intergenic	-	-	-	1512577	A	C	.	.	.	.	.	.	.	.	.	.	.	.	.	.	.	.	.	.	.	.	.	.	.	+
Intergenic	-	-	-	1516237	T	C	.	.	.	.	.	.	.	.	.	.	.	.	.	.	.	.	.	.	.	+	.	.	.	.
M5005_Spy1566	recD	S	1	1525835	A	G	.	.	.	.	.	.	.	.	.	.	.	.	.	.	.	.	.	+	.	.	.	.	.	.
M5005_Spy1571	-	N	-1	1532349	G	A	.	.	.	.	.	.	.	.	.	.	.	.	.	.	.	.	.	.	.	.	+	.	.	.
M5005_Spy1572	-	N	1	1533080	A	G	.	.	.	.	.	.	.	.	.	.	.	.	.	.	.	.	.	+	.	.	.	.	.	.
M5005_Spy1600	lppC	S	-1	1557859	G	A	.	.	.	.	.	.	.	.	.	.	.	.	.	.	.	.	.	.	.	.	.	.	.	+
M5005_Spy1602	-	S	-1	1560394	G	A	.	.	.	.	.	.	.	.	.	.	.	.	.	.	.	.	.	.	.	+	.	.	.	.
M5005_Spy1611	rpoE	S	-1	1568313	T	C	+	+	+	+	+	+	+	+	+	+	+	+	+	+	+	+	+	+	+	+	.	.	.	+
M5005_Spy1623	hsdM	S	1	1587031	C	T	.	.	.	.	.	.	.	.	.	.	.	.	.	.	.	.	.	.	.	.	+	.	.	.
Intergenic	-	-	-	1605161	G	T	.	.	.	.	.	.	.	.	.	.	.	.	.	.	.	.	.	.	.	.	.	+	+	.
Intergenic	-	-	-	1622205	G	A	.	.	.	.	.	.	.	.	.	.	.	.	.	.	.	.	.	+	.	.	.	.	.	.
M5005_Spy1665	-	N	-1	1622452	A	G	.	.	.	.	.	.	.	.	.	.	.	.	.	.	.	.	.	.	.	.	+	.	.	.
M5005_Spy1670	-	S	-1	1624684	A	G	+	+	+	+	+	+	+	+	+	+	+	+	+	+	+	+	+	+	+	+	+	+	+	+
M5005_Spy1671	-	N	-1	1625233	T	C	+	+	+	+	+	+	+	+	+	+	+	+	+	+	+	+	+	+	+	+	+	+	+	+
M5005_Spy1672	polC	S	-1	1625757	G	A	.	.	.	.	.	.	.	.	.	.	.	.	.	.	.	.	.	.	.	.	.	+	+	.
M5005_Spy1672	polC	S	-1	1629387	C	T	.	.	.	.	.	.	.	.	.	.	.	.	.	.	.	.	.	+	.	.	.	.	.	.
Intergenic	-	-	-	1629935	G	T	.	.	.	.	.	.	.	.	.	.	.	.	.	.	.	.	.	.	.	.	+	.	.	.
M5005_Spy1676	uppS	S	-1	1634935	C	T	.	.	.	.	.	.	.	.	.	.	.	.	.	.	.	.	.	.	.	+	.	.	.	.
M5005_Spy1679	-	N	-1	1636813	A	G	+	+	+	+	+	+	+	+	+	+	+	+	+	+	+	+	+	+	+	+	+	+	+	+
M5005_Spy1689	sclA	N	-1	1649243	T	C	+	+	+	+	+	+	+	+	+	+	+	+	+	+	+	+	+	+	+	+	+	+	+	+
M5005_Spy1689	sclA	N	-1	1649530	C	T	+	+	+	+	+	+	+	+	+	+	+	+	+	+	+	+	+	+	+	+	+	+	+	+

							Case 1						Case 2					Contacts				Other *emm* 1 isolates								
**Locus**	**Gene**	**S/N**	**Strand**	**Position**	**Ref base**	**SNP base**	BC‡	BC	Cx‡	GT¥	HH$ Contact	Baby	LRT‖	LRT	URT¶	URT	HH Contact	Doctor	Midwife	Nurse	Postpartum	Throat	Blood	Throat	Blood	Blood	Blood	Blood	Blood	Blood
Intergenic	-	-	-	1650800	C	T	.	.	.	.	.	.	.	.	.	.	.	.	.	.	.	.	.	.	.	.	+	.	.	.
M5005_Spy1704	dppA	N	1	1664313	G	A	.	.	.	.	.	.	.	.	.	.	.	.	.	.	.	.	.	.	.	.	+	.	.	.
M5005_Spy1704	dppA	N	1	1665132	G	A	.	.	.	.	.	.	.	.	.	.	.	.	.	.	.	.	.	.	.	.	+	.	.	.
M5005_Spy1718	sic	N	-1	1681676	A	C	+	+	+	+	+	+	+	+	+	+	+	+	+	+	+	.	.	.	.	.	.	.	.	.
M5005_Spy1719	emm1.0	S	-1	1682726	G	T	.	.	.	.	.	.	.	.	.	.	.	.	.	.	.	.	.	.	.	.	+	.	.	.
M5005_Spy1727	-	S	-1	1691722	C	T	.	.	.	.	.	.	.	.	.	.	.	.	.	.	.	.	.	.	.	.	+	.	.	.
M5005_Spy1730	-	N	-1	1694411	G	T	.	.	.	.	.	.	.	.	.	.	.	.	.	.	.	.	.	.	.	.	.	+	+	.
M5005_Spy1732	-	S	-1	1695410	C	G	.	.	.	.	.	.	.	.	.	.	.	.	.	.	.	.	.	.	.	.	.	+	.	.
M5005_Spy1737	rgg	N	1	1699526	G	T	.	.	.	.	.	.	.	.	.	.	.	.	.	.	.	.	.	.	.	+	.	.	.	.
M5005_Spy1737	rgg	N	1	1699901	A	G	+	+	+	+	+	+	+	+	+	+	+	+	+	+	+	+	+	+	+	+	+	+	+	+
M5005_Spy1738	spd	N	-1	1700243	G	A	.	.	.	.	.	.	.	.	.	.	.	.	.	.	.	.	.	+	.	.	.	.	.	.
M5005_Spy1738	spd	S	-1	1700687	T	C	.	.	.	.	.	.	.	.	.	.	.	.	.	.	.	.	.	.	.	.	.	+	+	.
M5005_Spy1753	pbp2A	N	-1	1713272	G	A	.	.	.	.	.	.	.	.	.	.	.	.	.	.	.	.	.	.	+	.	.	.	.	.
M5005_Spy1771	hutU	S	1	1732433	C	T	+	+	+	+	+	+	+	+	+	+	+	+	+	+	+	+	+	+	+	+	+	+	+	+
M5005_Spy1771	hutU	N	1	1734344	T	G	.	.	.	.	.	.	.	.	.	.	.	.	.	.	.	.	.	.	.	.	+	.	.	.
M5005_Spy1776	-	N	1	1739297	G	T	+	+	+	+	+	+	+	+	+	+	+	+	+	+	+	+	+	.	.	.	.	.	.	.
M5005_Spy1783	dexS	N	-1	1751016	C	A	.	.	.	.	.	.	.	.	.	.	.	.	.	.	.	.	.	.	+	.	.	.	.	.
M5005_Spy1788	yaaA	S	1	1757330	T	C	.	.	.	.	.	.	.	.	.	.	.	.	.	.	.	.	.	.	.	.	.	+	+	.
nrdD	-	S	-1	1760381	A	G	.	.	.	.	.	.	.	.	.	.	.	.	.	.	.	+	.	.	.	.	.	.	.	.
M5005_Spy1803	lmrP	S	-1	1770482	G	A	.	.	.	.	.	.	.	.	.	.	.	.	.	.	.	.	.	.	+	.	.	.	.	.
M5005_Spy1808	argS	N	1	1777467	A	G	.	.	.	.	.	.	.	.	.	.	.	.	.	.	.	.	.	.	.	+	.	.	.	.
M5005_Spy1808	argS	N	1	1777590	T	C	.	.	.	.	.	.	.	.	.	.	.	.	.	.	.	.	.	+	.	.	.	.	.	.
M5005_Spy1808	argS	N	1	1778151	G	A	+	+	+	+	+	+	+	+	+	+	+	+	+	+	+	+	+	.	.	.	.	.	.	.
M5005_Spy1819	-	S	1	1786938	A	G	.	.	.	.	.	.	.	.	.	.	.	.	.	.	.	.	.	.	.	.	.	+	+	.
M5005_Spy1830	-	N	1	1797058	G	A	.	.	.	.	.	.	.	.	.	.	.	.	.	.	.	.	.	.	.	+	.	.	.	.
Intergenic	-	-	-	1807270	C	A	.	.	.	.	.	.	.	.	.	.	.	.	.	.	.	.	.	.	.	.	.	+	.	.
M5005_Spy1840	trmU	S	-1	1807562	G	A	.	.	.	.	.	.	.	.	.	.	.	.	.	.	.	.	.	.	.	.	+	.	.	.
M5005_Spy1842	sdhA	S	1	1809717	T	C	+	+	+	+	+	+	+	+	+	+	+	+	+	+	+	+	+	+	+	+	+	+	+	+
M5005_Spy1852	hasB	N	1	1820674	C	A	.	.	.	.	.	.	.	.	.	.	.	.	.	.	.	.	.	.	.	.	.	.	.	+
M5005_Spy1852	hasB	N	1	1820841	T	C	.	.	.	.	.	.	.	.	.	.	.	.	.	.	.	.	.	.	.	+	.	.	.	.
M5005_Spy1853	hasC	S	1	1821821	T	C	+	+	+	+	+	+	+	+	+	+	+	+	+	+	+	.	.	.	.	.	.	.	.	.

							Case 1						Case 2					Contacts				Other *emm* 1 isolates								
**Locus**	**Gene**	**S/N**	**Strand**	**Position**	**Ref base**	**SNP base**	BC[†]	BC	Cx[‡]	GT[¥]	HH[$] Contact	Baby	LRT[‖]	LRT	URT[¶]	URT	HH Contact	Doctor	Midwife	Nurse	Postpartum	Throat	Blood	Throat	Blood	Blood	Blood	Blood	Blood	Blood
M5005_Spy1857	guaB	N	-1	1826109	A	G	.	.	.	.	.	.	.	.	.	.	.	.	.	.	.	.	.	+	.	.	.	.	.	.
M5005_Spy1861	-	N	1	1829839	G	A	.	.	.	.	.	.	.	.	.	.	.	.	.	.	.	.	.	.	.	.	+	.	.	.
M5005_Spy1862	-	S	1	1831522	T	C	.	.	.	.	.	.	.	.	.	.	.	.	.	.	.	.	.	.	.	.	+	.	.	.

[*]S/N; synonymous or nonsynonymous SNP
[†]BC; blood culture
[‡]Cx; Cervical isolate
[¥]GT; Genital tract
[‖]LRT; Lower respiratory tract
[¶]URT; Upper respiratory tract
[$]HH; household
Isolates are listed as they appear in Figure 2.

Table S3. Insertions and deletions identified in the core genomes of maternity unit isolates.

					Case 1						Case 2					Contacts			
**Locus**	**Gene**	**I/D[*]**	**Strand**	**Position**	BC[†]	BC	Cx[‡]	GT[¥]	HH[$] Contact	Baby	LRT[‖]	LRT	URT[¶]	URT	HH Contact	Doctor	Midwife	Nurse	Postpartum
M5005_Spy0041		D	1	63763	- T	- T	- T	- T	- T	- T	- T	- T	- T	- T	- T	- T	- T	- T	- T
Intergenic		I	-	155925	+ T	+ T	+ T	+ T	+ T	+ T	+ T	+ T	+ T	+ T	+ T	+ T	+ T	+ T	+ T
Intergenic		I	-	156051	+ G	+ G	+ G	+ G	+ G	+ G	+ G	+ G	+ G	+ G	+ G	+ G	+ G	+ G	+ G
M5005_Spy0194	gspA	I	-1	199430	+ G	+ G	+ G	+ G	+ G	+ G	+ G	+ G	+ G	+ G	+ G	+ G	+ G	+ G	+ G
M5005_Spy0283	covS	I	1	293203	+ T	+ T	+ T	+ T	+ T	+ T	+ T	+ T	+ T	+ T	+ T	+ T	+ T	+ T	+ T
Intergenic		D	-	317912	.	.	.	.	.	.	.	.	.	.	.	.	.	.	.
M5005_Spy0367	scaR	D	-1	371812	.	.	.	.	.	.	.	.	.	.	.	.	.	.	.
M5005_Spy0378	pyrH	D	1	381959	- A	- A	- A	- A	- A	- A	- A	- A	- A	- A	- A	- A	- A	- A	- A
M5005_Spy0378	pyrH	I	1	382108	+ T	+ T	+ T	+ T	+ T	+ T	+ T	+ T	+ T	+ T	+ T	+ T	+ T	+ T	+ T
Intergenic		D	-	447040	.	.	.	.	.	.	.	.	.	.	.	.	.	.	.
M5005_Spy0489		D	1	477225	- T	- T	- T	- T	- T	- T	- T	- T	- T	- T	- T	- T	- T	- T	- T
M5005_Spy0556	eno	I	1	547722	+ T	+ T	+ T	+ T	+ T	+ T	+ T	+ T	+ T	+ T	+ T	+ T	+ T	+ T	+ T
M5005_Spy0556	eno	D	1	547728	- A	- A	- A	- A	- A	- A	- A	- A	- A	- A	- A	- A	- A	- A	- A
Intergenic		D	-	575640	.	.	.	.	.	.	.	.	.	.	.	.	.	.	.
Intergenic		I	-	698058	.	.	.	.	.	.	.	.	.	.	.	.	.	.	.
M5005_Spy0731		I	1	734493	+ G	+ G	+ G	+ G	+ G	+ G	+ G	+ G	+ G	+ G	+ G	+ G	+ G	+ G	+ G
M5005_Spy0741	fbp	I	-1	744199	+ C	+ C	+ C	+ C	+ C	+ C	+ C	+ C	+ C	+ C	+ C	+ C	+ C	+ C	+ C
Intergenic		D	-	782326	- 5bp[#]	- 5bp	- 5bp	- 5bp	- 5bp	- 5bp	- 5bp	- 5bp	- 5bp	- 5bp	- 5bp	- 5bp	- 5bp	- 5bp	- 5bp
M5005_Spy0835		I	1	830120	+ A	+ A	+ A	+ A	+ A	+ A	+ A	+ A	+ A	+ A	+ A	+ A	+ A	+ A	+ A
M5005_Spy0850	pta	I	1	842477	+ G	+ G	+ G	+ G	+ G	+ G	+ G	+ G	+ G	+ G	+ G	+ G	+ G	+ G	+ G
Intergenic		I	-	900143	+ G	+ G	+ G	+ G	+ G	+ G	+ G	+ G	+ G	+ G	+ G	+ G	+ G	+ G	+ G
Intergenic		D	-	900731	- A	- A	- A	- A	- A	- A	- A	- A	- A	- A	- A	- A	- A	- A	- A
Intergenic		I	-	945555	+ C	+ C	+ C	+ C	+ C	+ C	+ C	+ C	+ C	+ C	+ C	+ C	+ C	+ C	+ C
M5005_Spy0966		D	-1	955755	- G	- G	- G	- G	- G	- G	- G	- G	- G	- G	- G	- G	- G	- G	- G
M5005_Spy1058	malE	I	1	1028134	+ C	+ C	+ C	+ C	+ C	+ C	+ C	+ C	+ C	+ C	+ C	+ C	+ C	+ C	+ C
Intergenic		I	-	1041428	.	.	.	.	.	.	.	.	.	.	.	.	.	.	.
M5005_Spy1118		I	-1	1092729	+ T	+ T	+ T	+ T	+ T	+ T	+ T	+ T	+ T	+ T	+ T	+ T	+ T	+ T	+ T
M5005_Spy1153		I	-1	1129115	+ C	+ C	+ C	+ C	+ C	+ C	+ C	+ C	+ C	+ C	+ C	+ C	+ C	+ C	+ C
Intergenic		I	-	1250753	+ C	+ C	+ C	+ C	+ C	+ C	+ C	+ C	+ C	+ C	+ C	+ C	+ C	+ C	+ C
Intergenic		D	-	1270407	- A	- A	- A	- A	- A	- A	- A	- A	- A	- A	- A	- A	- A	- A	- A
Intergenic		D	-	1350765	- T	- T	- T	- T	- T	- T	- T	- T	- T	- T	- T	- T	- T	- T	- T

Locus	Gene	I/D	Strand	Position	Case 1						Case 2					Screen			
					BC[ǂ]	BC	Cx[ǂ]	GT[¥]	HH[$] Contact	Baby	LRT[‖]	LRT	URT[¶]	URT	HH Contact	Doctor	Midwife	Nurse	Postpartum
M5005_Spy1537		D	-1	1495702	.	.	.	.	.	.	.	.	.	.	.	.	.	.	.
M5005_Spy1610	pyrG	I	-1	1567900	+ C	+ C	+ C	+ C	+ C	+ C	+ C	+ C	+ C	+ C	+ C	+ C	+ C	+ C	+ C
M5005_Spy1613		I	1	1570889	.	.	.	.	.	.	.	.	.	.	.	.	.	.	.
M5005_Spy1672	pulA	D	-1	1638919	- T	- T	- T	- T	- T	- T	- T	- T	- T	- T	- T	- T	- T	- T	- T
M5005_Spy1687	sclA	I	-1	1648751	.	.	.	.	.	.	.	.	.	.	.	.	.	.	.
M5005_Spy1689	sclA	D	-1	1649528	- A	- A	- A	- A	- A	- A	- A	- A	- A	- A	- A	- A	- A	- A	- A
M5005_Spy1693		I	-1	1653799	+ C	+ C	+ C	+ C	+ C	+ C	+ C	+ C	+ C	+ C	+ C	+ C	+ C	+ C	+ C
M5005_Spy1718	sic	D	-1	1681245	- 9bp	- 9bp	- 9bp	- 9bp	- 9bp	- 9bp	- 9bp	- 9bp	- 9bp	- 9bp	- 9bp	- 9bp	- 9bp	- 9bp	- 9bp
M5005_Spy1718	sic	D	-1	1681429	.	.	.	.	.	.	.	.	.	.	.	.	.	.	.
M5005_Spy1718	sic	D	-1	1681523	.	.	.	- 87bp	.	.	.	.	.	.	.	.	.	.	.
M5005_Spy1718	sic	D	-1	1681635	.	.	.	.	.	.	.	.	.	.	.	.	.	.	.
M5005_Spy1718	sic	D	-1	1681676	.	.	.	.	.	.	.	.	.	.	.	.	.	.	.
M5005_Spy1718	sic	D	-1	1681751	.	.	.	.	.	.	.	.	.	.	.	.	.	.	.
M5005_Spy1718	sic	D	-1	1681755	.	.	.	.	.	.	.	.	.	.	.	.	.	.	.
M5005_Spy1718	sic	D	-1	1681761	.	.	.	.	.	.	.	.	.	.	.	.	.	.	.
M5005_Spy1718	sic	D	-1	1681770	- 15bp	- 15bp	- 15bp	- 15bp	- 15bp	- 15bp	- 15bp	- 15bp	- 15bp	- 15bp	- 15bp	- 15bp	- 15bp	- 15bp	- 15bp
Intergenic		D	-	1695054	- T	- T	- T	- T	- T	- T	- T	- T	- T	- T	- T	- T	- T	- T	- T
M5005_Spy1829		I	-1	1796119	+ G	+ G	+ G	+ G	+ G	+ G	+ G	+ G	+ G	+ G	+ G	+ G	+ G	+ G	+ G
M5005_Spy1852	hasB	D	1	1820564	.	.	.	.	.	.	.	.	.	.	.	.	.	.	- A

[*]I/D; Insertion or deletion

[ǂ]BC; blood culture
[ǂ]Cx; Cervical isolate
[¥]GT; Genital tract
[‖]LRT; Lower respiratory tract
[¶]URT; Upper respiratory tract
[$]HH; household
[#]bp; base pair
Isolates are listed as they appear in Figure 2.

Table S4. Insertions and deletions identified in the core genomes of other *emm*1 isolates.

**Locus**	**Gene**	**I/D**[*]	**Strand**	**Position**	Throat	Blood	Throat	Blood	Blood	Blood	Blood	Blood	Blood
M5005_Spy0041		D	1	63763	- T	- T	- T	- T	- T	- T	- T	- T	- T
Intergenic		I	-	155925	+ T	+ T	+ T	+ T	+ T	+ T	+ T	+ T	+ T
Intergenic		I	-	156051	+ G	+ G	+ G	+ G	+ G	+ G	+ G	+ G	+ G
M5005_Spy0194	gspA	I	-1	199430	+ G	+ G	+ G	+ G	+ G	+ G	+ G	+ G	+ G
M5005_Spy0283	covS	I	1	293203	+ T	+ T	+ T	+ T	+ T	+ T	+ T	+ T	+ T
Intergenic		D	-	317912	.	.	.	- A	.	.	.	.	.
M5005_Spy0367	scaR	D	-1	371812	.	.	.	.	.	.	- T	- T	.
M5005_Spy0378	pyrH	D	1	381959	- A	- A	- A	- A	- A	- A	- A	- A	- A
M5005_Spy0378	pyrH	I	1	382108	+ T	+ T	+ T	+ T	+ T	+ T	+ T	+ T	+ T
Intergenic		D	-	447040	.	.	.	.	.	.	.	- T	.
M5005_Spy0489		D	1	477225	- T	- T	- T	- T	- T	- T	- T	- T	- T
M5005_Spy0556	eno	I	1	547722	+ T	+ T	+ T	+ T	+ T	+ T	+ T	+ T	+ T
M5005_Spy0556	eno	D	1	547728	- A	- A	- A	- A	- A	- A	- A	- A	- A
Intergenic		D	-	575640	.	.	.	- T	.	.	.	.	.
Intergenic		I	-	698058	.	.	.	.	+ A	.	.	.	.
M5005_Spy0731		I	1	734493	+ G	+ G	+ G	+ G	+ G	+ G	+ G	+ G	+ G
M5005_Spy0741	fbp	I	-1	744199	+ C	+ C	+ C	+ C	+ C	+ C	+ C	+ C	+ C
Intergenic		D	-	782326	- AAACA	- AAACA	.	.	.	.	.	.	.
M5005_Spy0835		I	1	830120	+ A	+ A	+ A	+ A	+ A	+ A	+ A	+ A	+ A
M5005_Spy0850	pta	I	1	842477	+ G	+ G	+ G	+ G	+ G	+ G	+ G	+ G	+ G
Intergenic		I	-	900143	+ G	+ G	+ G	+ G	+ G	+ G	+ G	+ G	+ G
Intergenic		D	-	900731	- A	- A	- A	- A	- A	- A	- A	- A	- A
Intergenic		I	-	945555	+ C	+ C	+ C	+ C	+ C	+ C	+ C	+ C	+ C
M5005_Spy0966		D	-1	955755	- G	- G	- G	- G	- G	- G	- G	- G	- G
M5005_Spy1058	malE	I	1	1028134	+ C	+ C	+ C	+ C	+ C	+ C	+ C	+ C	+ C
Intergenic		I	-	1041428	.	.	.	.	.	.	+ CTTTT	.	.
M5005_Spy1118		I	-1	1092729	+ T	+ T	+ T	+ T	+ T	+ T	+ T	+ T	+ T
M5005_Spy1153		I	-1	1129115	+ C	+ C	+ C	+ C	+ C	+ C	+ C	+ C	+ C
Intergenic		I	-	1250753	+ C	+ C	+ C	+ C	+ C	+ C	+ C	+ C	+ C
Intergenic		D	-	1270407	- A	- A	- A	- A	- A	- A	- A	- A	- A
Intergenic		D	-	1350765	- T	- T	.	.	.	.	- T	- T	.

Locus	Gene	I/D	Strand	Position	Throat	Blood	Throat	Blood	Blood	Blood	Blood	Blood	Blood
M5005_Spy1537		D	-1	1495702	.	.	.	.	.	.	- C	- C	.
M5005_Spy1610	pyrG	I	-1	1567900	+ C	+ C	+ C	+ C	+ C	+ C	+ C	+ C	+ C
M5005_Spy1613		I	1	1570889	.	+ A	.	.	.	.	.	.	.
M5005_Spy1672	pulA	D	-1	1638919	- T	- T	- T	- T	- T	- T	- T	- T	- T
M5005_Spy1687	sclA	I	-1	1648751	.	.	+ GTG	.	.	.	.	.	.
M5005_Spy1689	sclA	D	-1	1649528	- A	- A	- A	- A	- A	- A	- A	- A	- A
M5005_Spy1693		I	-1	1653799	+ C	+ C	+ C	+ C	+ C	+ C	+ C	+ C	+ C
M5005_Spy1718	sic	D	-1	1681245	- 9bp[#]	- 9bp	- 9bp	- 9bp	- 9bp	.	- 9bp	- 9bp	- 9bp
M5005_Spy1718	sic	D	-1	1681429	.	.	.	.	.	- 87bp	.	.	.
M5005_Spy1718	sic	D	-1	1681523	.	.	.	.	.	.	.	.	.
M5005_Spy1718	sic	D	-1	1681635	.	.	.	.	.	- 12bp	.	.	.
M5005_Spy1718	sic	D	-1	1681676	.	.	- 15bp	.	.	.	.	.	.
M5005_Spy1718	sic	D	-1	1681751	.	.	.	.	.	.	.	.	- 15bp
M5005_Spy1718	sic	D	-1	1681755	.	.	.	.	.	.	.	- 15bp	.
M5005_Spy1718	sic	D	-1	1681761	.	.	.	- 126bp	.	.	.	.	.
M5005_Spy1718	sic	D	-1	1681770	- 15bp	- 15bp	.	.	.	- 15bp	.	.	.
Intergenic		D	-	1695054	- T	- T	.	.	.	.	.	.	.
M5005_Spy1829		I	-1	1796119	+ G	+ G	+ G	+ G	+ G	+ G	+ G	+ G	+ G
M5005_Spy1852	hasB	D	1	1820564	.	.	.	.	.	.	.	.	.

[*]I/D; Insertion or deletion

[#]bp; base pair

Isolates are listed as they appear in Figure 2.

References

1. **Andrews JM**. 2001. Determination of minimum inhibitory concentrations. J Antimicrob Chemother. **48:**5-16.

2. **Lintges M, Arlt S, Uciechowski P, Plumakers B, Reinert RR, Al-Lahham A, Lutticken R, Rink L.** 2007. A new closed-tube multiplex real-time PCR to detect eleven superantigens of *Streptococcus pyogenes* identifies a strain without superantigen activity. Int J Med Microbiol. **297:**471-478.

3. **Mejia LM, Stockbauer KE, Pan X, Cravioto A, Musser JM.** 1997. Characterization of group A *Streptococcus* strains recovered from Mexican children with pharyngitis by automated DNA sequencing of virulence-related genes: unexpectedly large variation in the gene (*sic*) encoding a complement-inhibiting protein. J Clin Microbiol. **35:**3220-3224.

4. **Turner CE, Kurupati P, Jones MD, Edwards RJ, Sriskandan S.** 2009. Emerging Role of the Interleukin-8 Cleaving Enzyme SpyCEP in Clinical *Streptococcus pyogenes* Infection. J Infect Dis. **200:**555-563.

5. **Akesson P, Sjoholm AG, Bjorck L.** 1996. Protein SIC, a novel extracellular protein of *Streptococcus pyogenes* interfering with complement function. J Biol Chem. **271:**1081-1088.

6. **Kurupati P, Turner CE, Tziona I, Lawrenson RA, Alam FM, Nohadani M, Stamp GW, Zinkernagel AS, Nizet V, Edwards RJ, Sriskandan S.** 2010. Chemokine-cleaving *Streptococcus pyogenes* protease SpyCEP is necessary and sufficient for bacterial dissemination within soft tissues and the respiratory tract. Mol Microbiol. **76:**1387-1397.

7. **Schrager HM, Rheinwald JG, Wessels MR.** 1996. Hyaluronic acid capsule and the role of streptococcal entry into keratinocytes in invasive skin infection. J Clin Invest. **98:**1954-1958.

8. **Lancefield RC.** 1962. Current Knowledge of Type-Specific M antigens of group A *Streptococci*. J Immunol. **89:**307-313.

9. **Unnikrishnan M, Altmann DM, Proft T, Wahid F, Cohen J, Fraser JD, Sriskandan S.** 2002. The bacterial superantigen streptococcal mitogenic exotoxin Z is the major immunoactive agent of *Streptococcus pyogenes*. J Immunol. **169:**2561-2569.

10. **Li H, Durbin R.** 2009. Fast and accurate short read alignment with Burrows-Wheeler transform. Bioinformatics. **25:**1754-1760.

11. **Sumby P, Porcella SF, Madrigal AG, Barbian KD, Virtaneva K, Ricklefs SM, Sturdevant DE, Graham MR, Vuopio-Varkila J, Hoe NP, Musser JM.** 2005. Evolutionary origin and emergence of a highly successful clone of serotype M1 group A *Streptococcus* involved multiple horizontal gene transfer events. J Infect Dis. **192:**771-782.
